# Tracking alternative versions of the galactose gene network in the genus *Saccharomyces* and their expansion after domestication

**DOI:** 10.1016/j.isci.2024.108987

**Published:** 2024-01-23

**Authors:** Ana Pontes, Francisca Paraíso, Yu-Ching Liu, Savitree Limtong, Sasitorn Jindamorakot, Lene Jespersen, Carla Gonçalves, Carlos A. Rosa, Isheng Jason Tsai, Antonis Rokas, Chris Todd Hittinger, Paula Gonçalves, José Paulo Sampaio

**Affiliations:** 1 UCIBIO, Department of Life Sciences, Nova School of Science and Technology, Caparica 2829-516, Portugal; 2 Associate Laboratory i4HB, Nova School of Science and Technology, Caparica 2829-516, Portugal; 3 Biodiversity Research Center, Academia Sinica, Taipei 11529, Taiwan; 4 Department of Microbiology Faculty of Science, Kasetsart University, Bangkok 10900, Thailand; 5 Biodiversity Center Kasetsart University, Bangkok 10900, Thailand; 6 Microbial Diversity and Utilization Research Team, Thailand Bioresource Research Center, National Centre for Genetic Engineering and Biotechnology (BIOTEC), National Science and Technology, Development Agency (NSTDA), Pathum Thani 12120, Thailand; 7 Department of Food Science, University of Copenhagen, 1958 Frederiksberg C, Denmark; 8 Departamento de Microbiologia, ICB, C.P. 486, Universidade Federal de Minas Gerais, Belo Horizonte, MG 31270-901, Brazil; 9 Department of Biological Sciences, Vanderbilt University, Nashville, TN 37235, USA; 10 Evolutionary Studies Initiative, Vanderbilt University, Nashville, TN 37235, USA; 11 Laboratory of Genetics, DOE Great Lakes Bioenergy Research Center, Wisconsin Energy Institute, Center for Genomic Science Innovation, J.F. Crow Institute for the Study of Evolution, University of Wisconsin-Madison, Madison, WI 53726, USA

**Keywords:** Genetics, Genomics, Genotyping

## Abstract

When *Saccharomyces cerevisiae* grows on mixtures of glucose and galactose, galactose utilization is repressed by glucose, and induction of the *GAL* gene network only occurs when glucose is exhausted. Contrary to reference *GAL* alleles, alternative alleles support faster growth on galactose, thus enabling distinct galactose utilization strategies maintained by balancing selection. Here, we report on new wild populations of *Saccharomyces cerevisiae* harboring alternative *GAL* versions and, for the first time, of *Saccharomyces paradoxus* alternative alleles. We also show that the non-functional *GAL* version found earlier in *Saccharomyces kudriavzevii* is phylogenetically related to the alternative versions, which constitutes a case of *trans*-specific maintenance of highly divergent alleles. Strains harboring the different *GAL* network variants show different levels of alleviation of glucose repression and growth proficiency on galactose. We propose that domestication involved specialization toward thriving in milk from a generalist ancestor partially adapted to galactose consumption in the plant niche.

## Highlights

• Novel alternative *GAL* allele found in a wild population of *S. cerevisiae*
• Detection of alternative *GAL* alleles in *S. paradoxus* and *S. kudriavzevii*
• Glucose repression is relaxed or abolished in strains with alternative *GAL* genes
• Retention of *GAL* alternative alleles promoted adaptation to dairy environments

## Graphical abstract

**Figure:**
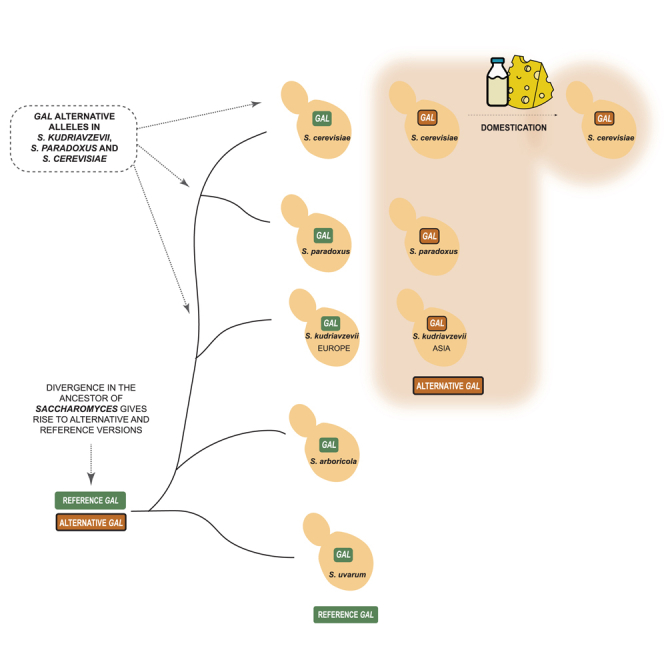

## Introduction

In nature, microorganisms typically thrive in complex and fluctuating environments and are faced with time-varying conditions that include the type of nutrients and specific stresses. Such habitat heterogeneity is understood to favor adaptive phenotypic plasticity, which can potentially lead to local adaptation.^1^^,^^2^ To cope with these challenges, biological systems evolved regulatory circuits that put in place diverse and elaborate cellular decision-making processes.^3^ With respect to nutrient utilization, the carbon catabolite repression systems are important and widespread regulatory mechanisms that prioritize the consumption of substrates that require the least resources and function by inhibiting pathways involved in the catabolism of carbon sources that are not preferred until the preferred ones are exhausted.^4^^,^^5^ Glucose is the preferred carbon source for the yeast *Saccharomyces cerevisiae*, as well as many other microorganisms.^6^^,^^7^^,^^8^^,^^9^ Importantly, the combination of this body of knowledge with recently acquired understanding of population structure in *S. cerevisiae*^10^^,^^11^^,^^12^ may foster the assessment of intra-specific phenotypic variation in the regulation of carbon metabolism and its contribution to adaptation in natural environments.

In complex and fluctuating environments, it is expected that optimization of ecological adaptation under a particular condition may have a fitness cost under other environmental conditions.^13^^,^^14^ Such evolutionary trade-offs have been well illustrated in *S. cerevisiae* growing on mixtures of carbon sources. Substrate transitions may cause growth to slow or stop for the period necessary to implement a new gene expression program suitable for the assimilation of the less preferred carbon source. The resulting growth profile is named diauxic and in such growth regimes, the overall fitness is positively correlated with the maximal growth rates on each of the carbon compounds and negatively correlated with the length of the transition phase.^15^ When *S. cerevisiae* is exposed to mixtures of glucose and galactose, galactose utilization is repressed by glucose and growth is typically diauxic. It is well known that the activation of the galactose (*GAL*) utilization pathway in this yeast is governed by a fine-tuned and strain-variable signal integration system that directs turning the *GAL* genes on or off, as well as determining their expression levels.^16^^,^^17^^,^^18^

The genetic makeup necessary for galactose metabolization in *S. cerevisiae* and many more species of the Saccharomycotina includes *GAL2* that codes for a galactose transporter and *GAL7*, *GAL10*, and *GAL1* that code for the enzymes that convert galactose into glucose 1-phosphate and that are the only genes of the network that are clustered. These four genes are usually designated the *GAL* structural genes. *PGM1* and *PGM2* are accessory genes not exclusively associated with galactose utilization that code for an isomerase that generates glucose 6-phosphate that subsequently enters glycolysis. This network also includes genes that code for a transcriptional activator (*GAL4*), a co-repressor (*GAL80*), and a co-inducer (*GAL3*).

In *S. cerevisiae*, more than 1,000 genome sequences are available,^12^ which opens the opportunity to access trait variation at the population level with an unprecedented detail. This led to the detection of distinct sets of *GAL* structural genes in certain populations. Besides the reference version, present in most strains and in the S288c reference genome, two alternative sets of highly divergent alleles of the *GAL* structural genes were recently uncovered.^19^^,^^20^ One such set of alleles, found in strains associated with dairy products,^11^^,^^12^^,^^20^^,^^21^ was found to support faster growth on galactose than on glucose, in sharp contrast to the reference alleles. The second set of alternative alleles was found in a few representatives of a wild Chinese population.^19^^,^^22^ *GAL* network recombination between reference and alternative alleles appears to be non-adaptative, which further highlights that the distinct galactose utilization strategies detected in *S. cerevisiae* appear to constitute an anciently established evolutionary trade-off maintained by a rare case of balancing selection acting on multiple loci instead of a single locus.^22^ This situation parallels the *GAL* network polymorphism maintained over a vast period of time in *S. kudriavzevii* and that results in Gal+ and Gal- populations.^23^

Here, we take advantage of the discovery of new wild populations of *S. cerevisiae* in Southeast Asia and in South America to revisit the diversification of the *GAL* gene network in the genus *Saccharomyces*. We uncover an additional alternative *GAL* version for *S. cerevisiae* and an alternative version for *S. paradoxus* that, together with previously known alternative versions in *S. cerevisiae* and the non-functional version found earlier in *S. kudriavzevii*, represent a remarkable case of *trans*-specific maintenance of a highly divergent *GAL* gene network that encompasses the entire genus. Domestication of *S. cerevisiae* in the dairy environment co-opted the alternative *GAL* version of the network, and we provide evidence that supports a phenotypic specialization toward thriving in milk from a more generalist one, which is partially adapted to galactose consumption in the plant niche.

## Results

### Alternative *GAL* alleles in *S. cerevisiae* and *S. paradoxus*

**Figure 1:**
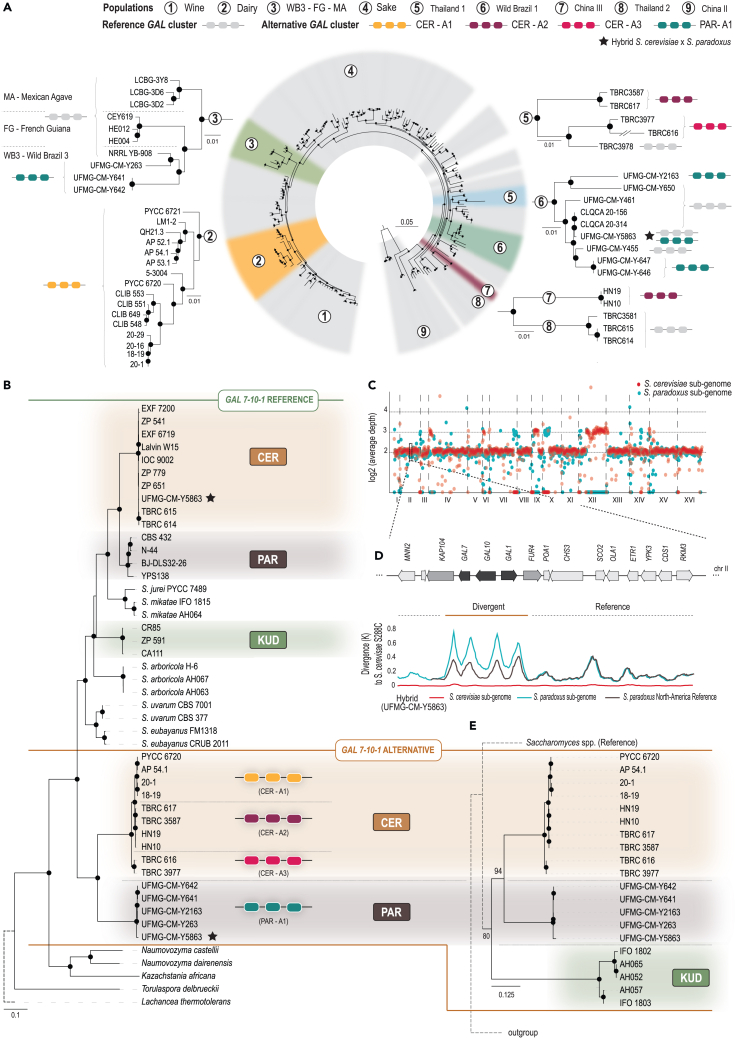
Reference and alternative versions of *GAL* genes are present in several *Saccharomyces* species and predate the formation of the genus (A) Phylogeny representing the known lineages of *S. cerevisiae* and highlighting in color those that harbor alternative *GAL* versions (reference version depicted in gray). In the case of populations 3 and 6 that harbor an alternative *GAL* version introgressed from *S. paradoxus* (see below), the reference version is also present as depicted in the pruned trees derived from whole genome data. For the Thailand 1 population, two alternative versions (CER-A2 and CER-A3) were detected together with the reference. The phylogeny was inferred from 214 sequences and 965,534 high-quality homozygous single nucleotide polymorphisms using the TVM+F+ASC+G model of sequence evolution and the maximum-likelihood method as implemented in IQ-TREE; it was rooted with representatives from the China IX/Taiwan 1 populations. Branch lengths correspond to the expected number of substitutions per site, and black dots in tree nodes depict bootstrap support values above 95% (1,000 replicates). (B) Concatenated maximum-likelihood phylogeny of the *GAL* gene cluster (*GAL7*-*10*-*1*) for the genus *Saccharomyces* showing the *trans*-specific organization of reference and alternative versions (CER, *S. cerevisiae*; PAR, *S. paradoxus*; KUD, *S. kudriavzevii*). The alternative *GAL* versions (CER-A1, CER-A2, CER-A3, and PAR-A1) are color coded. The phylogeny was rooted with *Lachancea thermotolerans*. (C) Whole-genome normalized coverage plot of the *S. cerevisiae* X *S. paradoxus* hybrid strain UFMG-CM-Y5863 showing the approximately equal contribution of both sub-genomes. Reads that mapped to *S. cerevisiae* are shown in red and those that mapped to *S. paradoxus* are shown in blue. (D) Divergence of the *S. cerevisiae* and *S. paradoxus* sub-genomes of UFMG-CM-Y5863 to *S cerevisiae* S288C in the region of chromosome II that includes the *GAL* cluster and several flanking genes. A third sequence, that of the American *S. paradoxus* strain YPS138, was used to control for the expected divergence between *S. cerevisiae* and *S. paradoxus.* Although the *S. cerevisiae* sub-genome contained the reference *GAL* version and showed almost no divergence, the *S. paradoxus* sub-genome diverged more than expected by comparison with the American *S. paradoxus* strain in the *GAL* region but not in the flanking regions. (E) Detail of a phylogeny of the *GAL* cluster including the alternative versions of *S. kudriavzevii*, which are all pseudogenes. In (D) and (E), black dots on the tree nodes indicate bootstrap values higher than 95% (2,000 replicates), and dotted lines represent branches not to scale. Scale bar corresponds to the expected number of substitutions per site.

Recently, a large dataset of 1,276 genome sequences of *S. cerevisiae* were surveyed for the presence of alternative *GAL* alleles.^22^ Here, we expanded this survey by analyzing representatives of two novel populations that we detected in Thailand and of two other populations previously found in Brazil^24^ (Table S1) but not yet investigated in detail. Our phylogenetic analysis comprised representatives of all known populations of *S. cerevisiae* described so far^11^^,^^12^^,^^21^^,^^25^ and included the recently described populations detected in Taiwan.^10^ The new Thai lineages, which we designate as Thailand 1 and Thailand 2, are entirely composed of strains isolated from natural environments (Table S1) and are closely related to other Asian lineages, namely Taiwan 2 (Thailand 1) and China III (Thailand 2). The survey of new genomes containing alternative *GAL* alleles allowed us to expand the inventory of alternative allelic versions from two to three, here designated as CER-A1, the alternative allele present in *S. cerevisiae* dairy strains; CER-A2, the alternative allele present in the China III population; and CER-A3, the newly found alternative allele detected in the Thailand 1 population (Figures 1A, 1B, and S2; Table S1). The first two alleles were previously found in strains associated with dairy products and in a wild Chinese population (China III),^19^^,^^22^ respectively. The novel one (CER-A3) was found in a wild Thai population (Thailand 1) that also harbored the CER-A2 and reference alleles (Figure 1A; Table S1). A phylogeny constructed based on an alignment of the concatenated *GAL7-10-1* genes of all natural (non-hybrid) *Saccharomyce*s species showed that all species have retained a reference *GAL* version and that the *S. cerevisiae* alternative versions are closely related with each other but nevertheless distinguishable (Figure 1B).

In addition, we obtained indirect evidence for the presence of an alternative *GAL* version in *S. paradoxus*, the closest relative of *S. cerevisiae*. This finding involved the study of a natural *S. cerevisiae* X *S. paradoxus* hybrid (strain UFMG-CM-Y5863) found in Brazil that had approximately equal contributions of the two species (Figure 1C). The existence of divergent *GAL* alleles in *S. paradoxus* can be seen in a sliding window analysis of the region of chromosome II that includes the *GAL* cluster (Figure 1D). In this analysis, we compared the divergence of the two sub-genomes of the hybrid and the divergence of an *S. paradoxus* sequence (strain YPS138 from North America) to *S. cerevisiae* S288c. Although the *S. cerevisiae* sub-genome contained the reference *GAL* version and showed almost no divergence to the reference *S. cerevisiae* genome, the *GAL7-10-1 S. paradoxus* sequence of the hybrid was considerably more divergent from the *S. cerevisiae* reference than the American *S. paradoxus* sequence (Figure 1D). The highly divergent region comprised only the *GAL* cluster, and divergence decreased sharply in the neighboring regions (Figure 1D). In line with this observation, the *S. paradoxus* sub-genome *GAL* sequence turned out to be phylogenetically more related to the alternative *GAL* versions of *S. cerevisiae* than to the reference versions of *S. cerevisiae* and remaining species in the genus (Figure 1B). This indirect finding of an alternative *GAL* version in *S. paradoxus* was corroborated by the detection of additional introgressed alternative *S. paradoxus GAL* versions in several Brazilian wild strains of *S. cerevisiae* (Figures 1A and 1B). The widespread dissemination of genetic material from *S. paradoxus* into the Brazilian populations WB1 and WB3 has been already reported,^24^ and the same phenomenon was observed for two other American populations of S. *cerevisiae*, usually designated as “French Guiana” and “Mexican Agavae,”^12^^,^^26^ and that are closely related to WB3, a wild Brazilian population (Figure 1A). Except for the 1:1 hybrid strain, all other strains harboring the *S. paradoxus* alternative version lacked the *S. cerevisiae* version. However, the alternative version seems to be absent among known isolates of *S. paradoxus* itself, as shown by the surveillance of more than 50 genomes from representatives of the various populations known, spanning Europe, Far East, China, and North America (Table S2).

Alternative versions of the structural gene *GAL2* were also previously detected in the Dairy and China III populations.^19^^,^^22^ In line with these findings, we also identified highly divergent versions of this gene in the newly found populations that harbored the alternative *GAL7*-*10*-*1* structural genes. The alternative *GAL2* genes were always duplicated, and, in case of dairy strains, the different copies resulted from recombination between reference and alternative alleles (Figure S1A). The phylogenetic placement of the remaining alternative versions was similar to what was observed for the *GAL* cluster (Figure S1B), suggesting that alternative *GAL2* genes have the same origin as alternative structural genes, as previously suggested.^22^ Interestingly, the Brazilian strains harboring *S. paradoxus GAL7-10-1* also carry *GAL2* genes whose phylogenetic placement is in line with an *S. paradoxus GAL2* alternative allele (Figure S1B). Moreover, the *Saccharomyces eubayanus GAL2* sequences that were recently reported as being divergent^27^ clustered at the base of the *S. eubayanus-Saccharomyces uvarum GAL2* (reference version) clade and not with the alternative versions (Figure S1B). This suggests that the *S. eubayanus* divergent versions are the remnants of a duplication that occurred on the reference version lineage; specifically, they occurred in the ancestor of *S. eubayanus* and *S. uvarum* and do not represent the alternative version. Another interesting finding was the detection of recombinant variants of *GAL7* in two strains of the African Beer population of *S. cerevisiae* (Figure S2A). Theses variants seem to result from the recombination of *S. cerevisiae* reference and alternative versions and have distinct contributions from each of the two versions (Figure S2B).

To further test if the diversification of the genes present in the cluster into reference and alternative versions predates the origin of the genus, we calculated the number of pairwise synonymous substitutions per synonymous sites (Ks). First, these measurements were made for the pair *S. cerevisiae*-*S. eubayanus* to have an estimate of divergence across the genus. Then, equivalent measurements were made between *S. cerevisiae* and *S. kudriavzevii* and, for an estimate of intraspecific divergence, between two *S. cerevisiae* strains from distinct populations. Then, these values were used as reference points to which estimates of *S. cerevisiae* and *S. paradoxus* reference versus alternative *GAL* cluster genes Ks estimates were compared. The Ks values calculated between reference and alternative *GAL* alleles were higher (Ks ∼1.5–∼2.5) than the value for *S. cerevisiae*-*S. eubayanus* (Ks∼1.1), which suggests that the divergence of the reference-alternative pairs is older than that of the genus *Saccharomyces* (Figure S3), as already reported for *S. cerevisiae*.^22^

### *GAL* pseudogenes of *S. kudriavzevii* correspond to the alternative *GAL* alleles

Using the *GAL7*-*10*-*1* sequences retrieved from the genomes of Asian *S. kudriavzevii* isolated in Taiwan, we observed that those genes appear to be phylogenetically more closely related to the alternative versions of *S. cerevisiae* and *S. paradoxus* than to the European version of *S. kudriavzevii* (Figures 1E and S4A). This result suggests that the European version corresponds to the reference version, and the Asian version might correspond to the alternative version of the *GAL* genes. Given that the Asian strains contain only *GAL* pseudogenes whose sequences are shorter than the normal sequences, we trimmed the remaining sequences in the alignment to the length of the corresponding pseudogenes for the phylogenies shown in Figures 1E and S4A. Despite their short lengths and degrees of pseudogenization, these sequences occupy positions in the phylogeny that suggest a shared evolutionary history with the *S. cerevisiae* and *S. paradoxus* alternative *GAL* version. We considered four tree topologies of alternative evolutionary scenarios and all yielded significantly lower likelihoods (Figure S4B, approximately unbiased test, p value < 0.05). In addition, we observed that the sequence divergence pattern is similar in the three species (*S. cerevisiae*, *S. paradoxus*, and *S. kudriavzevii*), with a parallel divergence built up in the flanking genes *KAP104* and *FUR4* (Figure S4C), which suggests a similar and ancient process of balancing selection in the three species. Furthermore, the *S. kudriavzevii GAL2* allele found in the Asian strains appears to correspond also to the alternative version, as sequences from these versions clustered with alternative versions of *S. cerevisiae* and *S. paradoxus*, albeit between these two species and with low statistical support (Figure S1C).

### Other genes related to galactose metabolism

**Figure 2:**
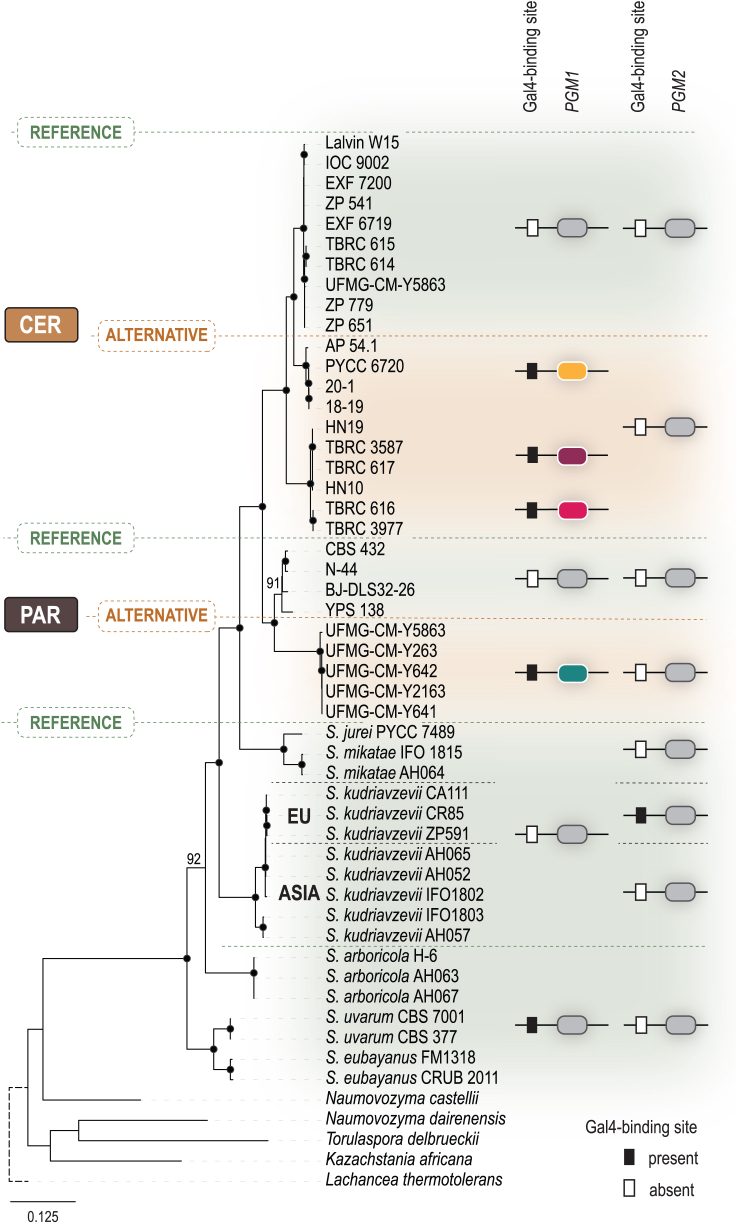
The duplication of *PGM1* occurred after speciation and only in *S. cerevisiae* and *S. paradoxus* Reference and alternative versions of *PGM1* are highlighted, together with presence or absence of the binding site for Gal4 in the promoter region of *PGM1* and *PGM2*. Maximum likelihood phylogeny inferred from 52 *PGM1* coding sequences using the GTR+F+I+G4 model of sequence evolution. The phylogeny was rooted with *Lachancea thermotolerans*, and the scale bar corresponds to the expected number of substitutions per site. Black dots on the tree nodes mark bootstrap values higher than 95% (2,000 replicates). The dotted line represents branches not to scale.

*PGM1*/*2* code for phosphoglucomutases that convert glucose-1-phosphate to glucose-6-phosphate, thereby connecting galactose metabolism with glycolysis. The genus-level phylogenetic reconstruction of the evolution of *PGM1* hinted at a trajectory distinct from that of the *GAL* cluster because a duplication of *PGM1* seems to have occurred more recently, only for *S. cerevisiae* and *S. paradoxus* and after speciation (Figure 2). Therefore, the *trans*-specific effect seen for the *GAL* cluster was not observed for *PGM1*. However, the number of alternative alleles we could recover was similar for the *GAL* cluster and *PGM1* in *S. cerevisiae* and *S. paradoxus* (Figure 2). For *S. kudriavzevii*, reference *PGM1* versions were found in the genomes that harbored the alternative and inactive *GAL7*-*10*-*1* version (Figure 2), contrary to *S. cerevisiae* and *S. paradoxus*.

It was shown that, in the Saccharomycetaceae, the promoter region of *PGM1*/*2* was the best predictor of robust growth on galactose and that improved growth was linked to the presence of one or more Gal4-binding site(s) in the promoter of either of the two paralogs.^28^ In *S. cerevisiae* strains with the *GAL* reference version, *PGM2* responds modestly to galactose through an unidentified mechanism independent of Gal4.^29^^,^^30^ More recently, it was shown that *S. cerevisiae* strains with alternative *GAL* alleles also harbored a Gal4-binding site in the *PGM1* promoter region, contrary to the strains carrying the reference *GAL* allele.^22^ In line with these findings, we observed that, in genomes with CER–A1, CER–A2, and CER–A3 alternative versions of *GAL7*-*10*-*1*, alternative versions of *PGM1* with one Gal4-binding site could also be found (Figure 2). Alternative *S. paradoxus PGM1* versions with promoters harboring Gal4-binding sites were also detected in *S. cerevisiae* strains carrying *S. paradoxus* alternative *GAL* alleles (Figure 2). In S. *kudriavzevii*, like in *S. cerevisiae GAL* reference strains,^31^ *PGM2* seems to be the most important of the two paralogs for galactose metabolism because *PGM2* harbors a functional Gal4-binding site in this species, although only in the European strains. However, no alternative versions of either *PGM1* or *PGM2* were found in *S. kudriavzevii*, and reference *PGM1* versions were found in genomes that harbored either the reference or the alternative and inactive *GAL7*-*10*-*1* version (Figure 2). Moreover, we detected the Gal4-binding site in *Saccharomyces arboricola, S. uvarum*, and *S. eubayanus*, three species for which we only found the reference version of *GAL7*-*10*-*1.* For *S. kudriavzevii*, *Saccharomyces jurei*, and *Saccharomyces mikatae*, the Gal4-binding site in the promoter region of *PGM1* was always absent. It is interesting to note that the alternative versions of *PGM1* always harbor the Gal4-binding site in their promoters (Figure 2; Table S1), an observation already made but for a more limited dataset.^22^

The *MEL1* gene is yet another Gal4-regulated gene. This gene codes for an α-galactosidase that hydrolyzes oligo- or disaccharides like raffinose or melibiose, which are natural sources of galactose. The *MEL1* gene is very rare in *S. cerevisiae*,^11^ and it was therefore striking to observe that it is present in most of the wild *S. cerevisiae* strains that possess the alternative *GAL* cluster (Table S1). This difference of *MEL1* occurrence in *GAL* reference and alternative wild backgrounds is statistically significant for the approximately 200 strains listed in Table S1 (t test, p value < 0.001).

### Growth rates on glucose and galactose

**Figure 3:**
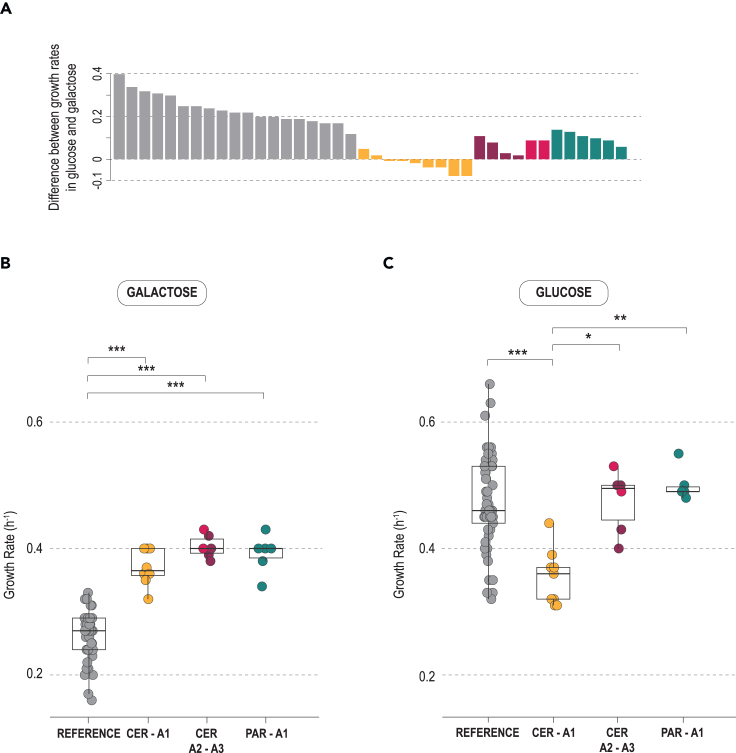
Comparison of growth rates in glucose and galactose among strains carrying the reference and alternative *GAL* versions (A) Bar plot depicting, for each strain, the difference between growth rate in glucose and growth rate in galactose in synthetic media supplemented with 2% (w/v) of carbon source. *GAL* allele types are color coded. For strains with the reference *GAL* allele, the results of a representative group are depicted, and full results are shown in Table S1. (B and C) Growth rates in galactose and glucose, respectively, measured using the same conditions as in (A). All groups were compared against each other with Dunn’s post hoc test. Levels of significance (p value): ∗∗∗0.001, ∗∗0.01, and ∗0.05.

It has been reported that strains with the alternative CER–A1 and CER–A2 alleles grow faster on galactose than strains with the reference alleles.^21^^,^^22^ Here, we expanded the analysis to include the novel alternative alleles presently uncovered. To this end, growth rates on glucose and galactose were first measured for strains harboring the reference allele. Those measurements varied considerably, as illustrated by the difference between growth rates on glucose and galactose (Figure 3A; Table S1), in line with previous studies.^18^^,^^19^ When the analysis was extended to strains with alternative versions, average growth rates on galactose were significantly higher for strains with alternative alleles in comparison with the reference *GAL* version (35%–50% higher; Kruskal-Wallis test, p value < 0.001; Figure 3B). On glucose, wild strains harboring alternative alleles (CER–A2, CER–A3, and PAR–A1) exhibited growth rates similar to those of strains carrying the reference *GAL* versions (Figure 3C), but domesticated strains carrying the CER–A1 allele had significantly lower median growth rates when compared with strains with the reference version (Kruskal-Wallis test, p value < 0.001), as previously observed.^19^

### Glucose repression of galactose utilization for alternative versus reference alleles

**Figure 4:**
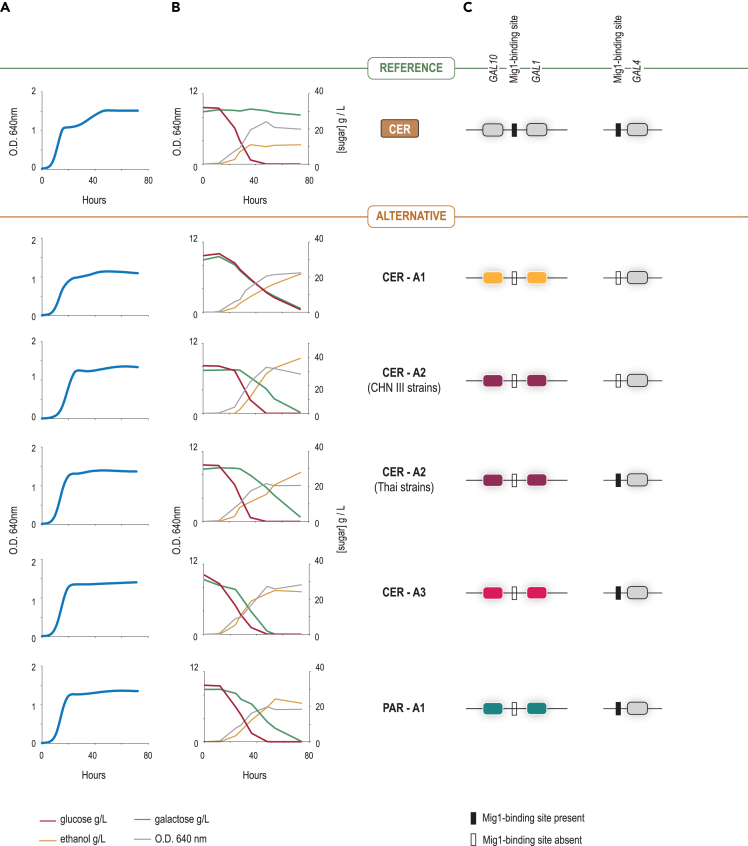
Strains with alternative *GAL* alleles have different levels of glucose repression (A) Growth curves in glucose and galactose medium (1% w/v each) of representative strains harboring either the reference or each of the various *GAL7-10-1* alternative alleles. (B) Profiles of glucose and galactose consumption, ethanol production, and growth for the strains shown in (A) in glucose and galactose medium (3% w/v each). Median values for all variables are shown from the replicas depicted in Figure S5. (C) Identification of presence or absence of Mig1-binding sites in the promoter regions of *GAL10*-7-*1* and *GAL4* across the spectrum of alternative versions.

Growth of *S. cerevisiae* on mixtures of glucose and other sugars usually leads to a diauxic growth pattern that reflects repression by glucose of the utilization of less favored carbon sources. Accordingly, growth was diauxic in strains harboring reference *GAL7-1-10* alleles growing on 96-well plates in medium containing 1% (w/v) each of glucose and galactose. However, none of the strains carrying any of the alternative alleles exhibited this growth pattern (Figure 4A). To confirm that this difference is due to a disruption in the usual pattern of sequential consumption of glucose and galactose, larger scale cultures were set up on medium containing equal amounts of glucose and galactose, and sugar consumption was quantified using HPLC. The results in Figures 4B and S5 show that, for strains carrying alternative *GAL7-1-10* alleles, the pattern of sugar consumption is clearly different from that observed for strains carrying the reference allele. Whereas in the latter case galactose consumption is initiated only after glucose is exhausted, strains with alternative versions consume glucose and galactose simultaneously or galactose consumption, although delayed, is initiated before glucose is totally consumed (Figures 4B and S5). For strains carrying the CER-A1 allele, it was previously reported that the combined effect of the lack of binding sites for the Mig1 repressor in the promoters of *GAL4* (activator of the *GAL* regulon) and of *GAL1-10*, combined with a mutation in the repressor *GAL80*, probably preventing its binding to Gal4, resulted in constitutive *GAL* structural gene expression in dairy strains.^19^ In addition, these strains lost the *HXT6* and *HXT7* genes, which negatively impacts glucose consumption rates. All these alterations resulted in co-consumption of glucose and galactose, with both sugars starting to be used at the same time and at very similar rates in dairy strains. The wild strains, in addition to having the Gal4-binding site in the *PGM1* promoter, also lacked a Mig1-binding site, either both in the *GAL* 1–10 promoter and the *GAL4* gene or only in the former. However, no relevant mutations were present in *GAL80* and *GAL3*. This gave rise to a pattern of sugar consumption in wild strains that was partially derepressed and was intermediate between the completely derepressed dairy strains and the fully repressed reference strains (Figure S5). Also, the extent of the delay of galactose assimilation relative to glucose and the rates of glucose consumption vary between the different alternative versions, raising the possibility that additional unidentified factors also impact the co-assimilation phenotype in strains carrying different classes of alleles. Because the *HXT6* and *HXT7* genes were present and seemingly functional in all wild strains, glucose transport is probably not responsible for differences in glucose consumption rates, although deficient expression of these transporters and/or the activity of other transporters cannot be excluded at this stage as possible explanations.

## Discussion

### Ancient origin and *trans*-specific distribution of *GAL* reference and alternative versions

Heretofore, the existence of highly divergent reference and alternative versions of *GAL* structural genes had only been demonstrated for *S. cerevisiae*.^19^ These versions have an ancient origin that predates the formation of the species and were maintained by balancing selection.^22^ Here, we show that a similar situation might exist in *S. paradoxus* as we detected an alternative *GAL* allele that groups closely to *S. cerevisiae* alternative *GAL* alleles. The increase in divergence seen in the two genes that flank *GAL7-1-10* is similar to what has been previously reported for the same genes in *S. cerevisiae*^22^ and *S. kudriavzevii*^23^ and supports the long-term maintenance of divergent states by balancing selection in the three species.

We did not detect the alternative alleles directly in existing *S. paradoxus* genomes but from *S. cerevisiae* X *S. paradoxus* hybrid or introgressed ones. We find it possible that the alternative *S. paradoxus* version is present in “clean” *S. paradoxus* genomes mainly for two reasons. First, the number of cultures and genome sequences available for this species is substantially lower than that of *S. cerevisiae*. Secondly, the frequency of the alternative version in *S. cerevisiae* is very low, especially in wild strains, as will be discussed below. Therefore, the relatively low number of *S. paradoxus* genomes surveyed is compatible with the current observations. Specifically, although we did not detect the alternative version in 50 genomes representative of the different populations of *S. paradoxus*, and another study that analyzed the *GAL* gene network of 108 *S paradoxus* genomes also did not report cases of high sequence divergence,^27^ more than 1,000 genomes have been inspected in *S. cerevisiae*. We suggest that the finding of the PAR-A1 allele only in introgressed or hybrid *S. cerevisiae* strains might be seen as accidental and not linked with the interspecies hybridization process. At this stage we see no reason, apart from poor sampling, for the absence of this allele in “clean” *S. paradoxus* strains, a situation that might change in the future.

In line with previous findings of incompatibility between alternative and reference *GAL* genes,^22^ our observations suggest the same level of genetic conflict in *S. paradoxus.* Only alternative *S. paradoxus GAL7-1-10*, *GAL2*, and *PGM1* were detected in Brazilian *S. cerevisiae* genomes retaining *S. paradoxus* introgressions. Moreover, our analyses in *S. kudriavzevii* suggest that the pseudogenized *GAL* alleles known in this species^23^^,^^32^ correspond to the alternative versions of *S. cerevisiae* and *S. paradoxus*.

Together, these findings support a scenario of an ancient divergence of *GAL* alleles in the ancestor of the genus *Saccharomyces* that become *trans*-specific through multiple speciation events, including the most recent one, that gave rise to *S. cerevisiae*. The MHC locus in mammals is a well-known case of an ancient *trans*-specific polymorphism that spans species boundaries.^33^ Although an introgression from an unknown species could, conceptually, yield the observed results, forward simulations models rejected the alternative possibilities of neutral introgression or introgression followed by maintenance of the alternative and reference alleles with multi-locus balancing selection.^22^ Given the most likely scenario of ancient divergence, the absence of the alternative version in most of the existing *Saccharomyces* species remains to be explained. We searched the available genomes of *S. mikatae*, *S. jurei*, *S. arboricola*, *S. uvarum*, and *S. eubayanus*, but we only found the reference versions of *GAL7*-*10*-*1.* Moreover, a recent study that included an expanded genome dataset of all species of the genus *Saccharomyces* also did not report additional divergent *GAL* alleles, except for divergent *GAL2* alleles,^27^ which, as our results show, were more closely related to the reference version. As already discussed for *S. paradoxus*, it remains to be elucidated if the alternative version has been lost in these species or if it has not yet been found, as genome sampling in *Saccharomyces* species other than *S. cerevisiae* is considerably lower.

For *S. cerevisiae*, the disproportion between the frequencies of reference and alternative *GAL* alleles is notorious. From more than 1,300 genomes that have been examined, approximately 50 (less than 4%) contain the alternative versions. Our findings in *S. paradoxus*, in which the alternative version was only indirectly found, and in *S. kudriavzevii*, in which the alternative version is non-functional, suggest that the reference version is currently more advantageous and hint at the alternative version being on the verge of extinction. It is however remarkable that the mechanisms that in the past favored the maintenance of the two versions acted on a similar way in multiple species. We hypothesize that the large population sizes and the wide ranges of *Saccharomyces* species, together with their heterogeneous environments, favored the retention of extensive polymorphisms such as the *GAL* multi-locus divergence. Such polymorphisms would be hard to maintain in macroscopic organisms with smaller population sizes and experiencing, by virtue of their size and smaller number, more homogeneous environments.

### Ecological relevance of galactose

**Figure 5:**
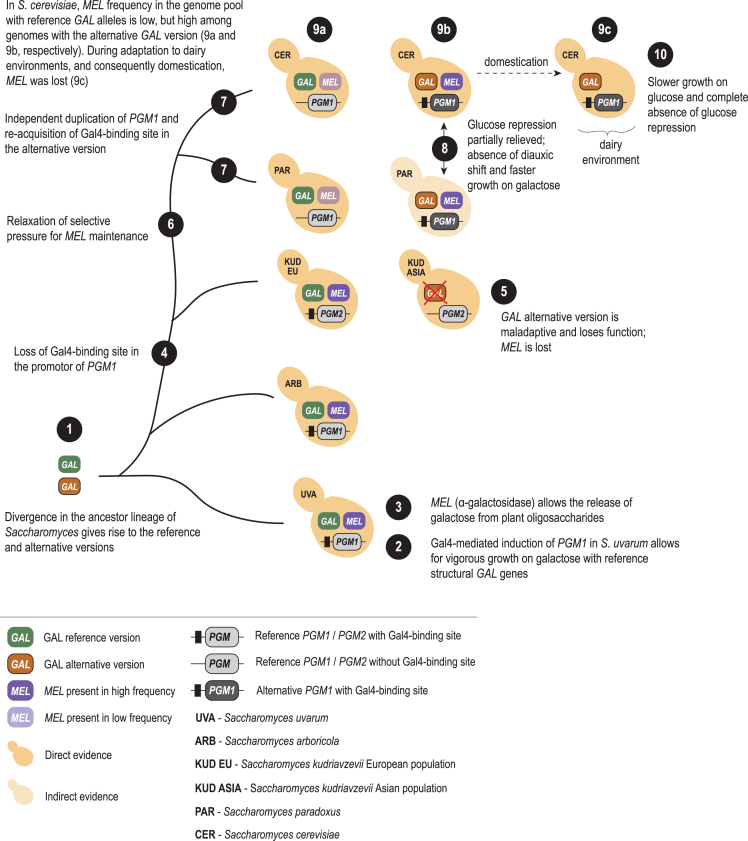
A model of the functional evolution of *GAL* reference and alternative versions across the genus *Saccharomyces.*

Galactose is frequently associated with milk and milk products, which are a direct consequence of livestock domestication.^34^^,^^35^ However, this sugar is also a common building block of many oligosaccharides of plant origin that constitute natural galactose stores.^36^^,^^37^ For example, whereas the trisaccharide raffinose (galactose, glucose, and fructose) contains one galactose moiety, stachyose, verbascose, and ajugose contain 2, 3, and 4 galactose moieties, respectively. Thus, galactose of plant origin is ubiquitous^38^^,^^39^ and can exceed glucose when released from plant oligosaccharides.^40^ Given the primal association of *Saccharomyces* to the arboreal niche,^41^^,^^42^^,^^43^ it is conceivable that efficient galactose utilization was among the favored traits present in the ancestor of the genus. For example, *S. uvarum*, an early derived species in the genus, has an active *GAL* gene network^39^^,^^40^^,^^44^ that, among other features, includes the induction of *PGM1* by galactose^28^ (Figure 5). We postulate that a more generalist strategy toward sugar utilization is an ancestral trait in the genus and that, gradually, a specialist glucose-prioritizing lifestyle gained prevalence, with *S. cerevisiae* being the epitome of this ecological behavior (Figure 5).

### Phenotypic plasticity at the population level

Evolution of galactose utilization in budding yeasts is remarkably dynamic, particularly with respect to regulatory mechanisms.^29^ Moreover, in *S. cerevisiae,* various studies have contributed to reveal an extensive phenotypic plasticity of galactose utilization strategies,^16^^,^^17^^,^^18^ in which the strains carrying the alternative *GAL* alleles^19^^,^^22^ represent one of the ends of this spectrum. Here, we detected an additional version of an alternative *GAL* allele (CER-A3) in a wild Thai population that also harbored the CER-A2 alternative allele and the reference one. The coexistence of alternative and reference alleles in the same population or in groups of populations that share the same environment, like in the case of Chinese sympatric wild populations, can be seen as a strategy to maximize ecological fitness in a changing environment.^45^ Given that selection maintains the alternative and reference alleles, it is conceivable that, in the habitats explored by these lineages, the coexistence of cells with different phenotypes is adaptive, which would allow for a more thorough exploration of the nutritional landscape. Thus, by maintaining cells with different optimization programs, a population could explore both low and high galactose concentrations, which can be seen as a case of niche complementation^46^ that maximizes optimality under a particular condition and reduces adjustment between conditions.^3^^,^^47^ However, even if this was the reason that promoted the maintenance of reference and alternative alleles, their asymmetric representation in *S. cerevisiae* and *S. paradoxus* and inactivation in *S. kudriavzevii* (Figure 5) might be an indication that the selective advantages of maintaining the alternative *GAL* system have diminished over time. Alternatively, it is of relevance to note that the maintenance of the two states by balancing selection does not require that their frequencies are similar. Finally, the reason behind the loss of the alternative version by gene inactivation in *S. kudriavzevii* might be particular to this species, given that the two versions are spatially separated at a continental scale.^23^^,^^27^

### Functional evolution of *GAL* reference and alternative alleles

Numerous studies have analyzed the function and regulation of the (reference) *GAL* network in *S. cerevisiae*.^7^^,^^16^^,^^17^^,^^48^ Overall, this network encodes a regulatory program of slow induction and strong repression of galactose utilization by glucose. This program leads to a fitness gain when glucose is abundant but to a fitness defect during transition from glucose to galactose.^48^ By contrast, *S. uvarum* is more active toward galactose utilization and has better induction and less-stringent repression mechanisms.^28^^,^^44^^,^^49^ Even in *S. paradoxus*, the closest relative of *S. cerevisiae*, the activity of the (reference) *GAL* network is initiated at a much lower galactose concentration in comparison with what is observed in *S. cerevisiae*.^50^ Given that these distinct phenotypes are based on reference *GAL* versions (Figure 5), we reason that subordination to glucose consumption was not present in the last common ancestor of *S. cerevisiae* and *S. uvarum* but evolved subsequently in the lineage that led to *S. cerevisiae*. Moreover, this glucose specialization appears to have been counterbalanced by the maintenance of an alternative *GAL* version.

### Domestication recovered the alternative and rare *S. cerevisiae GAL* version

The alternative *GAL* version seen in a few wild *S. cerevisiae* populations was captured and become fixed during the domestication of the dairy lineage. This domestication event illustrates the strong power of artificial selection in seizing an almost extinct genetic variant and, paradoxically, in contributing to increase the phenotypic diversity of the species. Moreover, artificial selection in the dairy environment selected for phenotypes that were considerably distinct from those of wild strains harboring the alternative *GAL* version and thriving in the plant niche. Specialization toward galactose utilization and to a more constant environment completely abolished glucose repression in the dairy lineage, even though it is still present in their ancestor wild strains. Domestication also resulted in poor growth on glucose and in the loss of *MEL* genes (Figure 5).

The transition from the plant niche to the dairy environment during microbe domestication appears to be a common feature and encompasses other yeasts,^51^ filamentous fungi in cheeses,^52^^,^^53^ and lactic acid bacteria in milk.^54^^,^^55^ Similarly, to what we observed here, strong metabolic specialization toward the milk environment transformed plant niche generalists into dairy specialists.

### Limitations of the study

Although one of the goals of this research was to search for the presence of alternative versions of the *GAL* gene network among the species of the genus *Saccharomyces*, this task was not fully accomplished because some species have been poorly sampled. Given the observed low frequency of detection of alternative versions among well-represented species, inferences for taxa with low numbers of isolates are difficult to make.

## STAR★Methods

### Key resources table

**Table:** 

REAGENT or RESOURCE	SOURCE	IDENTIFIER
**Chemicals, peptides, and recombinant proteins**		

Yeast Nitrogen Base	BD	Cat#239210
D-(+)-Glucose	Sigma-Aldrich	Cat#G8270; CAS no: 50-99-7
D-(+)-Galactose	Sigma-Aldrich	Cat# G0750, CAS no: 59-23-4
Ethanol	Honeywell	Cat#32221; CAS no: 64-17-5
D-(+)-Raffinose pentahydrate	Sigma-Aldrich	Cat#83400; CAS no: 17629-30-0
D-(+)-Glucosamine hydrochloride	Sigma-Aldrich	Cat#G4875; CAS no: 66-84-2

**Critical commercial assays**		

MiSeq Reagent Kit v2 (500-cycles)	Illumina	MS-102-2003
Nextera XT	Illumina	FC-131-1096
NextSeq 500/550 Mid Output Kit v2 (300 cycles)	Illumina	20024905

**Deposited data**		

Whole Genome sequencing data	DDBJ/ENA/GenBank	PRJEB63270
*GAL*/*PGM* gene sequence	DDBJ/ENA/GenBank	OR393451-OR393572

**Experimental models: Organisms/strains**		

See Table S1 for list of strains	This paper	N/A

**Software and algorithms**		

GATK	Depristo et al.^56^	https://gatk.broadinstitute.org/hc/en-us
BWA (v.0.7.17)	Li et al.^57^	https://github.com/lh3/bwa
Picard (v.2.22.8)	Github	http://broadinstitute.github.io/picard/
IQ-TREE (v.16.11)	Nguyen et al.^58^	http://www.iqtree.org/
MAFFT (v7.407)	Katoh et al.^59^	https://github.com/DomBennett/om..mafft
catfasta2phyml	Github	https://github.com/nylander/catfasta2phyml
iTOL (v5)	Letunic et al.^60^	https://itol.embl.de/
SPAdes (v3.13.1)	Bankevich et al.^61^	https://github.com/ablab/spades
trimmomatic (v0.36)	Bolger et al.^62^	https://github.com/usadellab/Trimmomatic
variscan (v2.0)	Vilella et al.^63^	http://www.ub.edu/softevol/variscan/
sppIDER (v1)	Langdon et al.^64^	https://github.com/GLBRC/sppIDer
YEASTRACT+	Monteiro et al.^65^	http://www.yeastract.com/
UGENE v43.0	Okonechnikov et al.^66^	https://github.com/ugeneunipro/ugene
Growthcurver	Sprouffske et al.^67^	https://cran.r-project.org/web/packages/growthcurver/index.html

**Other**		

P680 HPLC Pump	Dionex	N/A
ASI 100 Automated Sample Injector	Dionex	N/A
Tecan Spark Microplate reader	Tecan Trading	N/A

### Resource availability

#### Lead contact

Further information and requests for resources and reagents should be directed to and will be fulfilled by the Lead Contact, José Paulo Sampaio (jss@fct.unl.pt).

#### Materials availability

No unique reagents were generated in this study.

#### Data and code availability

• Whole-genome data has been deposited in DDBJ/ENA/GenBank and is publicly available as of the date of publication. Accession numbers are listed in the key resources table. The sequences of the *GAL*/*PGM* genes of the strains isolated in Taiwan have been deposited in GenBank and are publicly available as of the date of publication. Accession numbers are listed in the key resources table.
• This paper does not report original code.
• Any additional information required to reanalyze the data reported in this paper is available from the lead contact upon request.

### Experimental model and study participant details

*Saccharomyces cerevisiae* strains used in this study are listed in Table S1. The selected strains were grown in YNB supplemented either with 0.2% (w/v) glucose, 2% (w/v) of either glucose or, galactose –or with 1%–3% (w/v) glucose +1%–3% (w/v) of galactose. All growth experiments were performed at 25°C with no agitation.

### Method details

#### Genome sequencing, read alignment, and genotype calling

DNA was extracted from overnight grown cultures and paired-end Illumina MiSeq (500 cycles) and NextSeq (300 cycles) reads were obtained. Additional genomic data used in this study were retrieved from public databases as indicated in Table S1.

Single nucleotide polymorphisms (SNPs) from whole-genome sequencing data were extracted following an adapted GATK germline short variant discovery pipeline.^68^ Reads from each genome were mapped to a combined reference genome that included *S. cerevisiae* S288c (version R64-1-1) and *S. paradoxus* CBS 432 (ASM207905v1) using BWA v.0.7.17.^57^ Duplicated reads were marked with Picard v.2.22.8 (http://broadinstitute.github.io/picard/). SNPs, INDELS (insertions and deletions), and genotype determination were performed on all samples simultaneously using a local re-assembly of haplotypes (using GATK HaplotypeCaller, Genomics DBImport, and Genotype GVCFs). Standard hard filtering parameters of variant quality score recalibration were adjusted according to GATK best practice recommendations (GATK VariantFiltration with parameter values QD < 2.0, QUAL <30.0, SOR >3.0, FS > 60.0, MQ < 40.0, MQRankSum < −12.5, ReadPosRankSum < −8.0).^56^

#### Phylogenetic analyses and survey of specific genes

The whole-genome phylogeny was inferred using the maximum-likelihood method as implemented in IQ-TREE v.16.11,^58^ using the best-fit model of sequence evolution (TVM+F+ASC+G4) and ultrafast bootstrap approximation with 1,000 replicates.^59^ Strains from the populations CHN IX and Taiwan 1 were used to root the tree based on previous studies.^10^

For gene phylogenies, nucleotide sequences were aligned with MAFFT v7.407.^59^ The aligned sequences were used to reconstruct a maximum-likelihood phylogeny using IQ-TREE v.16.11,^58^ the best-fit model of sequence evolution inferred by the software (-m TEST), and ultrafast bootstrap with 2,000 replicates. For the concatenated phylogeny of the *GAL* gene cluster (*GAL7-10-1*) the same strategy was used but, after the alignment of individual genes, the PERL script catfasta2phyml (https://github.com/nylander/catfasta2phyml) was used to concatenate the files. To reconstruct the phylogeny with the concatenated file, a partition flag (-spp) and a file with information on the partitions was used in the command line of IQ-TREE. The software iTOL v5^60^ was used for all tree visualizations. Tree topology tests were performed using an approximately unbiased (AU) test^69^ in IQ-TREE v.16.11^58^ against the original phylogeny.

Specific genes were investigated in whole-genome *de novo* assemblies generated with SPAdes v3.13.1.^61^ Prior to assembly, reads were processed with trimmomatic v0.36^62^ to remove adapter sequences. In order to retrieve genes of interest, a local BLAST database was created for each genome and ORFs of interest were searched with blastn using query sequences from *GAL7*, *GAL10*, *GAL1*, *GAL2*, *GAL3*, *GAL4*, *GAL80*, and *PGM1* retrieved from the SGD database; the *MEL1* sequence was retrieved from strain UWOPS 03–461.4.^70^ Annotations of *S. cerevisiae* strain Lalvin W15 and ZP 779 were transferred from the S288c reference using liftoff v.6.1.^71^

#### Divergence analysis and hybrid identification

Divergence of the *GAL* gene cluster and flanking regions was estimated using VARISCAN v2.0^63^ for each selected strain by comparison with the reference genome of *S. cerevisiae* using RunMode 21. The results were processed using a 1,000- bp sliding window with 100- bp step increments. For smaller regions (analyses of individual genes), the same approach was used with a 100- bp sliding window with 20- bp step increments. For detection and analysis of hybrid genomes and introgressions, the program spider v1^64^ was used. Single copy orthologs between *S. cerevisiae* strain Lalvin W15 and ZP 779, *S. cerevisiae* Lalvin W15 – *S. eubayanus* FM1-318 and *S. cerevisiae* Lalvin W15 – *S. kudriavzevii* ZP 591 species pairs were inferred from the proteomes using Orthofinder v2.5.4^72^ and aligned using CLUSTALW v2.1.^73^ Synonymous substitutions (Ks) of *Saccharomyces* species ortholog-pair or *S. cerevisiae* strain gene-pair were calculated from the alignments using PAML v4.10.6.^74^

#### Search for binding sites of transcription factors

The searches for the Gal4-binding site motif (CGGN_11_CCG) in the *PGM1* promoter region and for the Mig1-binding site (SYGGRG) in the promoter regions of the *GAL4* and *GAL1/GAL10* genes were performed using YEASTRACT+.^65^ For the Mig1-binding sites, the results were manually curated using UGENE v43.0^66^ to verify and retrieve sequences.

#### Growth assays and sugar consumption determination

Cultures were pre-grown in 200 μL of yeast nitrogen base (YNB) medium (Difco) supplemented with 0.2% (w/v) glucose in a 96-well plate overnight at 25°C. Then, 5 μL of the cultures were inoculated in 200 μL of YNB supplemented with 2% (w/v) of either, glucose or, galactose –or with 1% (w/v) glucose +1% (w/v) of galactose. In all cases, at least two independent experiments were performed, growth was followed by measuring the absorbance at 640 nm for 72 h in a microplate reader (Tecan Spark, Tecan Trading, Männedorf, Switzerland) at 25°C. Growth rates and carrying capacity were calculated using the R package Growthcurver.^67^

For measurements of glucose and galactose in mixtures of both sugars, cultures were pre-grown overnight in YNB with 0.2% (w/v) glucose at 25°C. Cultures were then inoculated in 50 mL of YNB with 3% (w/v) glucose +3% (w/v) galactose, at an initial optical density 640nm of 0.015 and incubated at 25°C for 74 h. Growth was monitored and samples were harvested at various time points for glucose, galactose, and ethanol quantification. Samples were analyzed by high-performance liquid chromatography (HPLC) using a carbohydrate analysis column (300 mm by 7.8 mm, Aminex HPX-87P; Bio-Rad) and a differential refractometer (Shodex R-101). The column was kept at 80°C, and water was used as the mobile phase at 0.6 mL min^−1^. To account for strain variability, two independent experiments and measurements were performed for at least two representative strains of each *GAL* allele.

### Quantification and statistical analysis

Statistical analyses were performed in RStudio with custom scripts under available packages. The Kruskal-Wallis and t-test were performed in R using kruskal.test and t.test function (respectively), after testing for the normality of the data with the Shapiro-Wilk normality test (shapiro.test). For all statistical tests, ‘∗∗∗’ corresponds to a p value of 0.001, ‘∗∗’ p value 0.01, and ‘∗’ p value 0.05.
