# Supplementary material for: Tracking alternative versions of the galactose gene network in the genus Saccharomyces and their expansion after domestication
Source: iScience. 2024 Jan 23;27(2):108987. doi: 10.1016/j.isci.2024.108987 (PMC10850751; doi:10.1016/j.isci.2024.108987)
Supplement: Document S1. Figures S1–S5 and Tables S1 and S2 [file mmc1.pdf]

iScience, Volume *27*

Supplemental information

Tracking alternative versions of the galactose gene network in the genus *Saccharomyces* and their expansion after domestication

Ana Pontes, Francisca Paraíso, Yu-Ching Liu, Savitree Limtong, Sasitorn Jindamorakot, Lene Jespersen, Carla Gonçalves, Carlos A. Rosa, Isheng Jason Tsai, Antonis Rokas, Chris Todd Hittinger, Paula Gonçalves, and José Paulo Sampaio

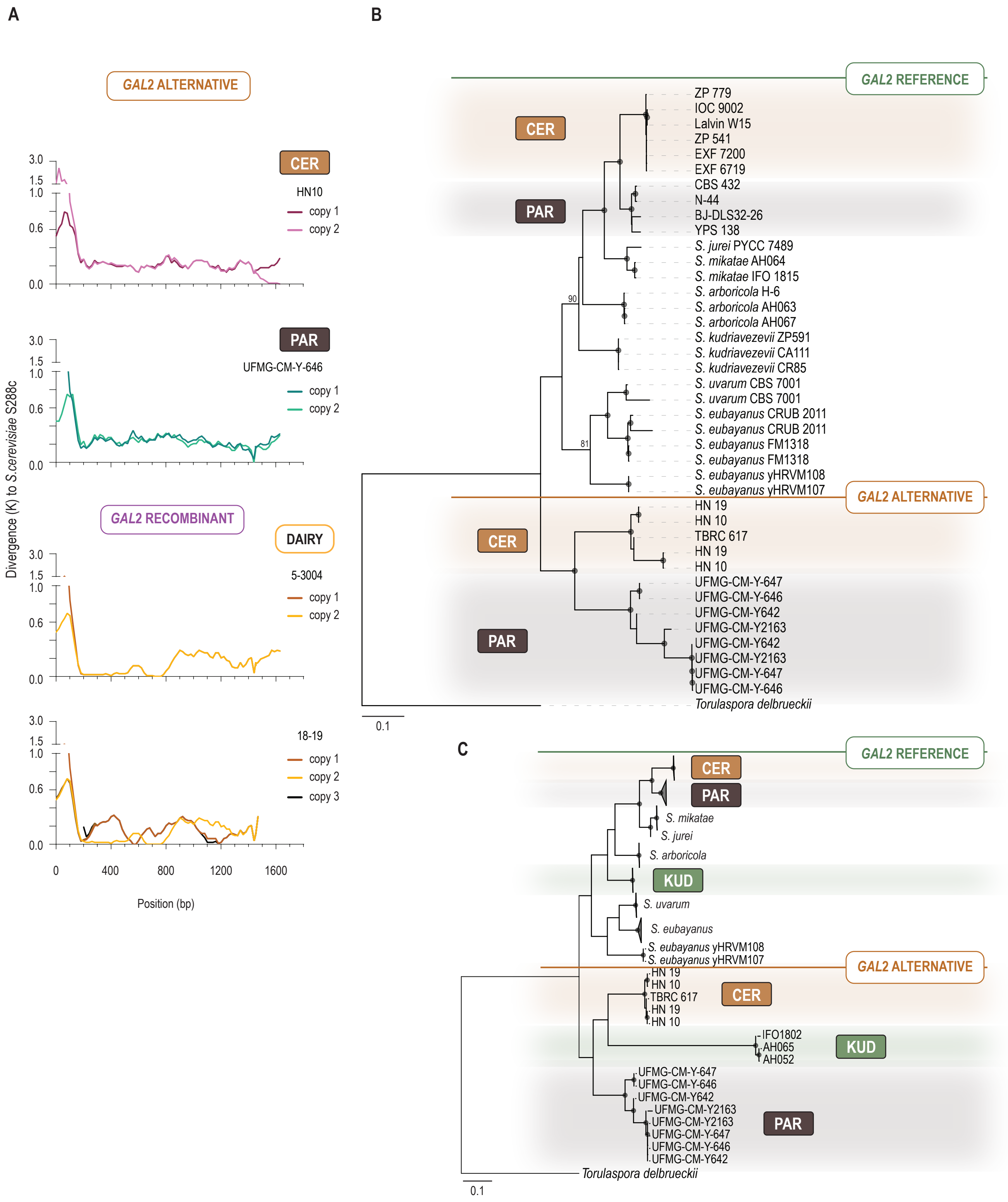

Figure S1. *GAL2* trans-specific polymorphism and recombination, related to Figure 1. (A) Divergence plots of *GAL2* for alternative versions of *S. cerevisiae* (HN10), *S. paradoxus* (UFMG-CM-Y-646), and *S. cerevisiae* dairy recombinant versions (5-3004 and 18-19). Divergence was calculated against the *S. cerevisiae* S288C reference. **(B)** Maximum- likelihood phylogeny inferred from 41 sequences using the TVM+F+I+G4 model of sequence evolution. The phylogeny was rooted with *Torulaspora delbrueckii*, and the scale bar corresponds to the expected number of substitutions per site. Black dots on the tree nodes mark bootstrap values higher than 95% (2,000 replicates). Reference and alternative versions are indicated (*S. cerevisiae*, CER; *S. paradoxus*, PAR). **(C)** Maximum-likelihood phylogeny inferred from 44 sequences partial sequences of *GAL2*. The alignment was trimmed by the length of the pseudogenes present in the Asian strains of *S. kudriavzevii*. The phylogeny was constructed as in (b) with the HKY+F+G4 model of sequence evolution (*S. kudriavzevii*, KUD).

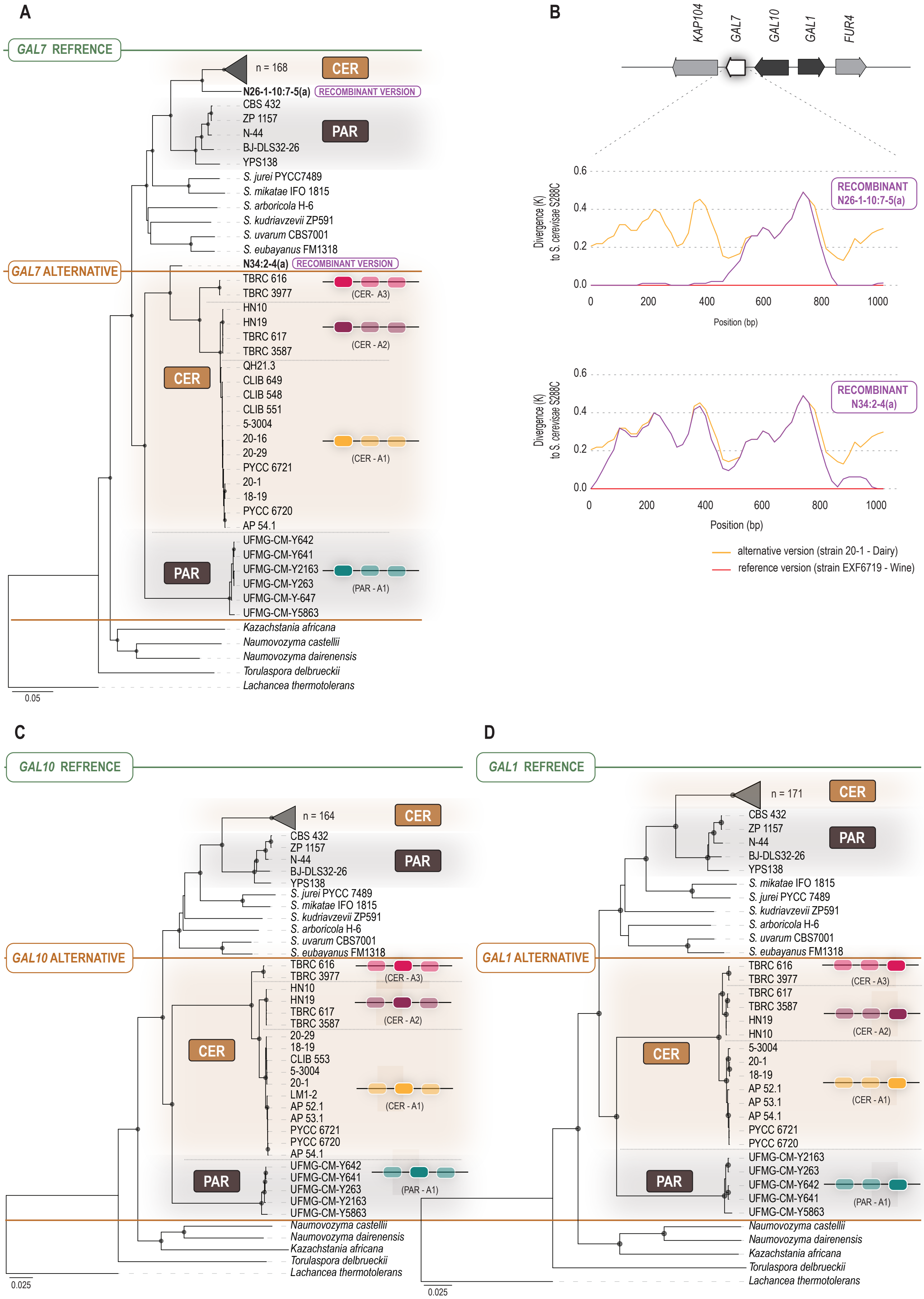

Figure S2. Individual *GAL7-10-1* phylogenies and evidence of recombination, related to Figure 1. (A) Phylogenetic tree of *GAL7* inferred from 210 complete sequences using the TIM3+F+I+G4 model of sequence evolution and the maximum-likelihood method. The phylogeny was rooted with *Lachancea thermotolerans*, black dots on the tree nodes mark bootstrap values higher than 95% (2,000 replicates), and the scale bar represents substitutions per site. *S. cerevisiae* reference version sequences are collapsed, reference and alternative versions are highlighted, and recombinant sequences are marked in bold. **(B)** Divergence plot of *GAL7* for the recombinant African beer strains N26-1-10:7-5(a) and N34:2-4(a) calculated against *S. cerevisiae* reference strain (S288C). A strain from the Dairy population and a strain from the Wine population are also included to show the expected *GAL7* divergence for alternative and reference versions, respectively. **(C and D)** Phylogenetic tree of *GAL10* (C) and *GAL1* (D) inferred from 202 and 206 complete sequences, respectively, using the TIM3+F+I+G4 model of evolution and the maximum-likelihood method. Other details are as in A.

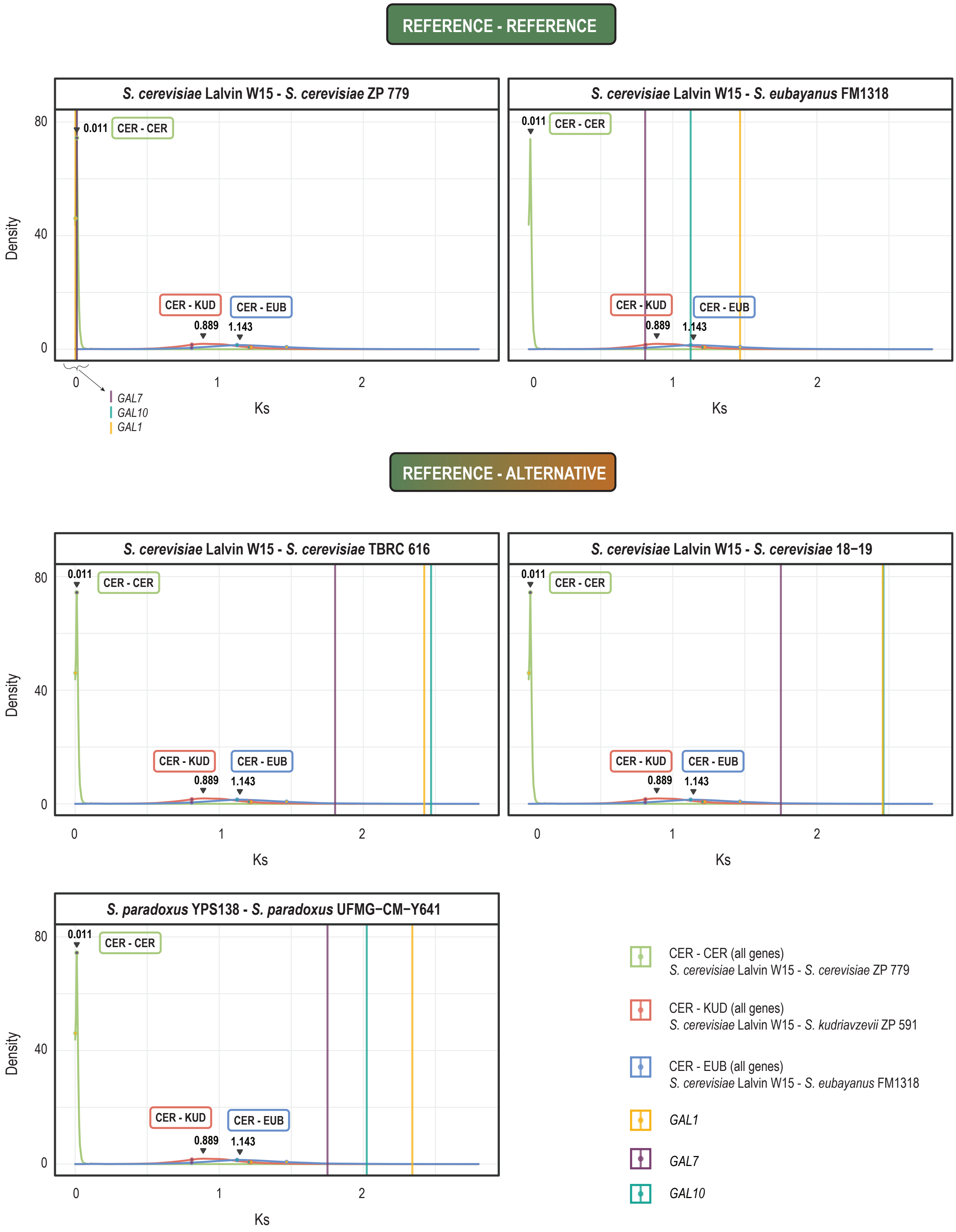

Figure S3. Density plots of synonymous substitutions (Ks) of *GAL* alternative versions point to an origin that predates the genus, related to Figures 1 and 5. Density plots for whole genome pairwise comparisons of Ks for *S. cerevisiae – S. cerevisiae*, *S. cerevisiae – S. kudriavzevii* and *S. cerevisiae – S eubayanus*. Ks was also calculated for the *GAL* cluster genes, considering pairs of strains with the reference allele and pairs of strains with reference and alternative alleles (CER, *S. cerevisiae*; KUD, *S. kudriavzevii*; EUB, *S. eubayanus*).

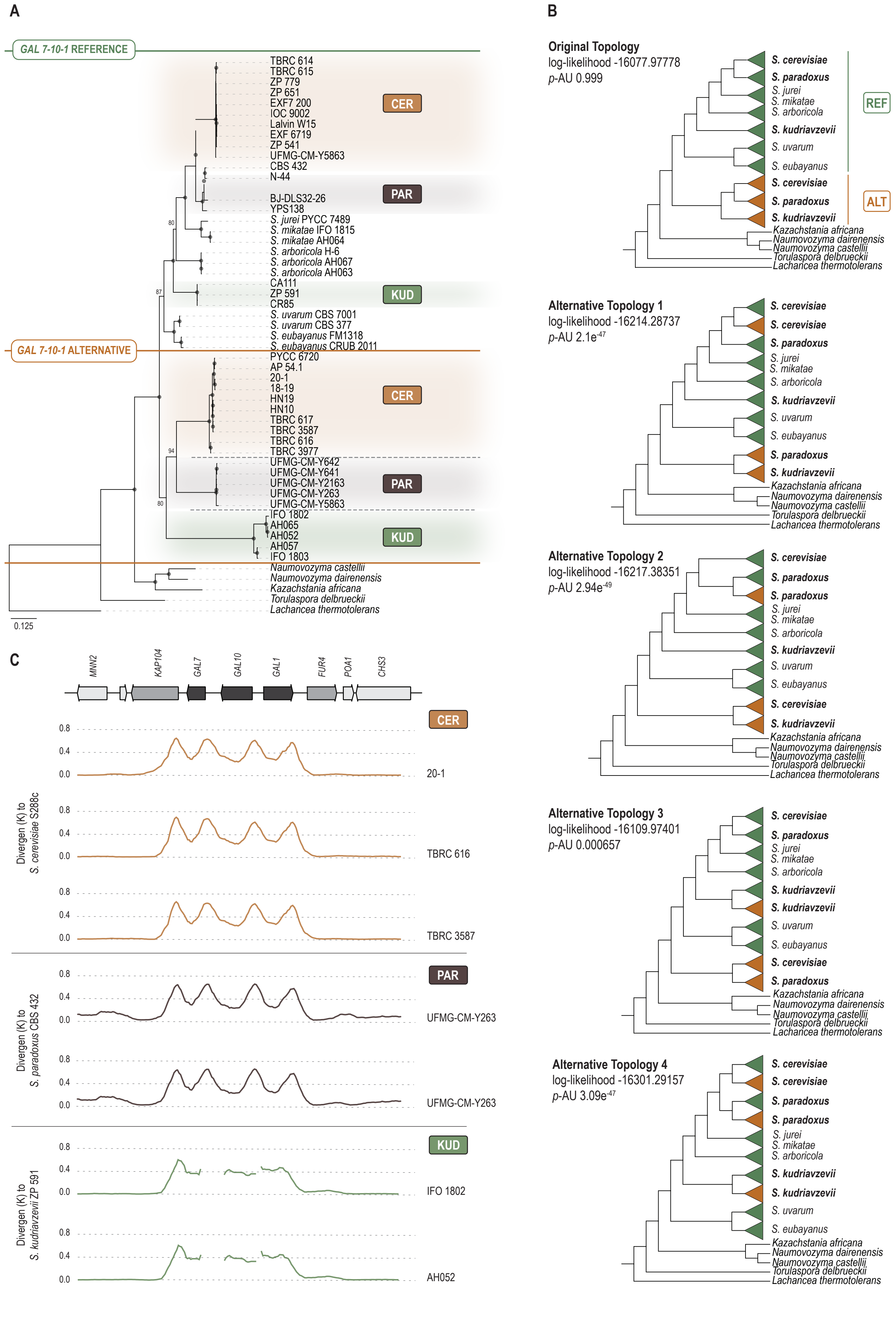

Figure S4. *S. kudriavzevii GAL* pseudogenes correspond to the alternative version, related to Figure 1. (A) Concatenated maximum-likelihood phylogeny of the partial sequences of the *GAL* gene cluster (*GAL7-10-1*) for the genus *Saccharomyces*. The phylogeny was constructed using the TIM3+F+G4 model of sequence evolution and was rooted with *Lachancea thermotolerans*. Black dots on the tree nodes mark bootstrap values higher than 95% (2,000 replicates), and the scale bar represent substitutions per site. High bootstrap values, but lower than 95, are depicted by their absolute value. **(B)** Topology test using the dataset presented in (a). Four alternative topologies consider that the alternative *GAL* versions emerged more recently than in the original topology. Reference versions are represented in green, and alternative versions in brown. Log-likelihood and *p*-AU values are indicated for each topology. **(C)** Divergence of the alternative *GAL* gene cluster and flanking regions for representative strains of each species against the respective reference genome (CER, *S. cerevisiae*; PAR, *S. paradoxus*; KUD, *S. kudriavzevii*).

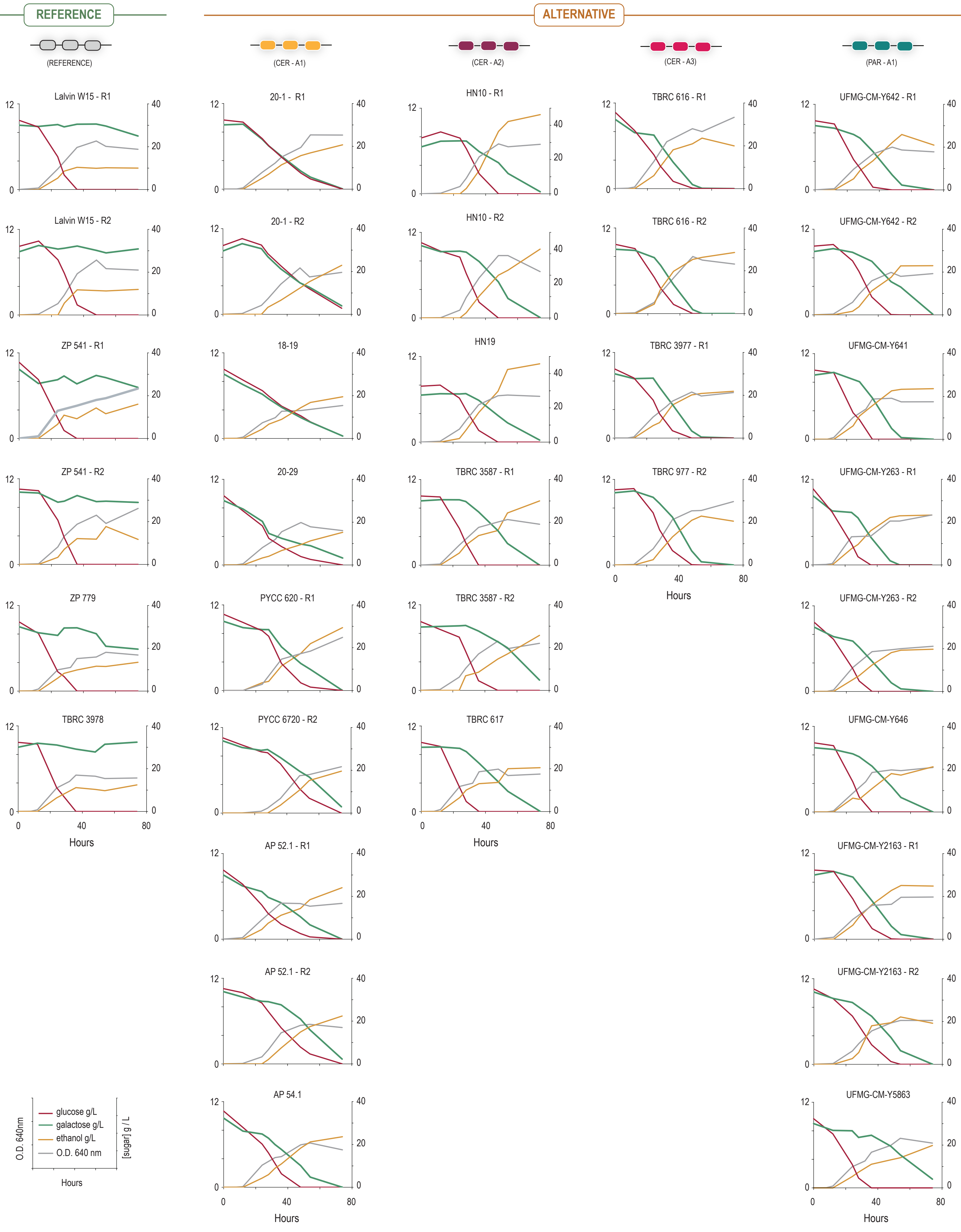

Figure S5. Growth curves on glucose and galactose and consumption profiles, related to Figure 4. Profiles of glucose and galactose consumption, ethanol production, and growth in 3% glucose + 3% galactose medium for an expanded group of strains containing either the reference or each of the various *GAL7-10-1* alternative alleles. Strains for which more than one experiment was made are represented by R1 (replica 1) and R2 (replica 2).

TableS1

Supplementary Table 1. Strains and genomes used in this study and relevant information pertaining to them, related to Figures 1, 2, 3 and 4.

Strain	Other Designations	WGS Group	Substrate	Geographic Location	GENOTYPE: GAL Cluster *GAL7*	*GAL10*	*GAL1*	*GAL2*	*PGM1*	UAS *GAL4*	Mig1-binding site: Prom. *GAL 4*	Prom. *GAL1/10*	*MEL*	PHENOTYPE: 1% Glucose + 1% Galactose: Diauxic Growth	Growth Rate	Carrying Capacity	2% Galactose: Growth Rate	Carrying Capacity	2% Glucose: Growth Rate	Carrying Capacity	Growth Rate 2% Glucose - 2% Galactose	Genome Data	Reference
5-18	CAA	WINE - PDM	Wine conserved in amphora	Georgia				1		x	✓	✓	x									ERP014555	[S1]
PYCC 6729		WINE - PDM	Jerez-wine	Armenia				1		x	✓	✓	x	--	--	--	NO GROWTH		0.48	0.92	--	PRJEB19382	[S2]
CBS 4054	AES	WINE - PDM	Red wine	Spain				1		x	✓	✓	x									ERP014555	[S1]
CBS 6006	AQE	WINE - PDM	Wine	Spain				1		x	✓	✓	x									ERP014555	[S1]
EC 1118		WINE - PDM	Industrial strain isolated from Champagne	France				1		x	✓	✓	x									PRJEA37863	[S3]
Lalvin QA23		WINE - PDM						1		x	✓	✓	x									PRJNA48561	[S4]
IOC 18-2007		WINE - PDM	Commercial yeast	Institut Oenologique de Champagne, France				1		x	✓	✓	x	--	--	--	NO GROWTH		0.40	1.14	--	PRJEB19382	[S2]
PYCC 4074		WINE - PDM	commercial wine yeast (Fermichamp)	Portugal				1		x	✓	✓	x	--	--	--	NO GROWTH		0.33	1.20	--	PRJEB19382	[S2]
ICO9002		WINE	wine (commercial strain)	Mêda, Guarda, Portugal				1		x	✓	✓	x	✓	--	1.37	0.26	1.39	0.43	1.09	0.17	SRP059414	[S5]
PYCC 8196	CBS 1460	WINE		Indonesia				1		x	✓	✓	x	✓	--	1.32	0.23	1.39	0.46	1.10	0.23	PRJEB36095	[S6]
Lalvin W15		WINE	Commercial yeast (white and red wine)					1		x	✓	✓	x	✓	--	1.45	0.24	1.41	0.46	1.22	0.22	PRJEB19382	[S2]
Uvaferm SGV		WINE						1		x	✓	✓	⊠									PRJEB19382	[S2]
EXF 6719		WINE	Wine must	Cuber, Slovenia				1		x	✓	✓	x	✓	--	1.15	0.24	1.41	0.55	1.17	0.31	PRJEB7601	[S5]
PYCC 6727	CBS 1227	WINE	Human skin infection	France				1		x	✓	✓	x	✓	--	1.39	0.20	1.43	0.39	1.22	0.19	PRJEB36095	[S6]
CBS 5635	AHN	WINE	Grape must	South Africa				1		x	✓	✓	✓									ERP014555	[S1]
ZP 641		WINE	Spontaneous red wine fermentation	Castelo de Vide, Portugal				1			✓		x									PRJEB7601	[S5]
Lalvin BM45		WINE	Commercial yeast (white wine)	Italy				1		x	✓	✓	x									PRJEB19382	[S2]
UFMG-CM-Y030	CAY1007	CACHAÇA	Cachaça (commercial strain)	Minas Gerais, Brazil				1		x	✓	✓	✓	✓	--	1.34	0.29	1.33	0.45	1.02	0.16	PRJEB24932	[S7]
UFMG-CM-Y630	SC3	CACHAÇA	Cachaça	Santa Catarina, Brazil				1		x	✓	✓	x	✓	--	1.08	0.16	1.45	0.47	1.04	0.31	PRJEB24932	[S7]
CBS 7959		CACHAÇA	Factory producing ethanol (sugar cane syrup)	Sao Paulo, Brazil				1		x	✓	✓	x									ERP014555	[S1]
UFMG-CM-Y633	T09	CACHAÇA	Cachaça	Tocantins, Brazil				1		x	✓	✓	x	✓	--	1.11	0.21	1.47	0.42	1.22	0.21	PRJEB24932	[S7]
UFMG-CM-Y634	TOC 1301	CACHAÇA	Cachaça	Tocantins, Brazil				1		x	✓	✓	x	--	--	--	NO GROWTH		0.32	1.16	--	PRJEB24932	[S7]
PYCC 2608	CBS 1782	BEER 2	Beer Type of *Saccharomyces diastaticus*					1		x	✓	✓	x									PRJEB36095	[S6]
TUM 1-B-8		BEER 2	spoilage yeast Isolated from brewery	Germany, State Bavaria (Bayern)				1		x	✓	✓	x									PRJEB36095	[S6]
TUM PI-BB-105		BEER 2	spoilage yeast Isolated from beer-mixed beverage	Unknown				1		x	✓	✓	x									PRJEB36095	[S6]
TUM 71		BEER 2	spoilage yeast Isolated from brewery	Germany, State North-Rhine Westphalia (Nordrhein-Westfalen)				1		x	✓	✓	✓									PRJEB36095	[S6]
AP 52.1		DAIRY	Camel Fermented Milk (dough)	Jandagh, Iran				1		✓	x	x	x	x	0.38	1.20	0.40	1.06	0.39	0.98	-0.01	PRJEB63270	This sudy
AP 53.1		DAIRY	Cow Fermented Milk (dough)	Roudbar, Iran				3		✓		x	x	x	0.40	0.86	--	--	0.44	0.83	--	PRJEB63270	This sudy
AP 54.1		DAIRY	Cow Fermented Milk (dough)	Semnan, Iran				2		✓	x	x	x	x	0.35	1.16	0.36	1.06	0.37	1.11	0.02	PRJEB63270	This sudy
CLIB 649	BGV	DAIRY	Dairy, whey	Alpes, France				1		✓	x		x									ERP014555	[S1]
CLIB 548	BFV	DAIRY	Dairy, cheese (camembert)	Normandy, France				1					x									ERP014555	[S1]
CLIB 551	BGC	DAIRY	Dairy, cheese (camembert)	Normandy, France						✓			x									ERP014555	[S1]
CLIB 553	BGE	DAIRY	Dairy, raw milk	Normandy, France						✓	x	x	x									ERP014555	[S1]
20-1		DAIRY	Acidified milk 'nunu'	Northern Ghana, Africa				3		✓	x	x	x	x	0.32	1.10	0.40	0.98	0.32	0.95	-0.08	PRJEB63270	This sudy
20-29		DAIRY	Acidified milk 'nunu'	Northern Ghana, Africa						✓	x	x	x	x	0.36	1.13	0.37	1.03	0.36	1.04	-0.01	PRJEB63270	This sudy
20-16		DAIRY	Acidified milk 'nunu'	Northern Ghana				1		✓	x		x	x	0.32	1.08	0.40	0.90	0.32	1.05	-0.08	PRJEB63270	This sudy
18-19		DAIRY	Acidified milk 'nunu'	Northern Ghana, Africa				4		✓	x	x	x	x	0.29	1.09	0.36	0.99	0.31	0.93	-0.04	PRJEB63270	This sudy
QH21-3		DAIRY	Fermented yak milk	Delingha, Qinghai				2			x	x	x									PRJNA396809	[S8]
LM1-2		DAIRY	Fermented yak milk	Naqu, Tibet				2			x		x									PRJNA396809	[S8]
5-3004		DAIRY	Fermented mare's milk (kumis)	Ulaanbaatar, Mongolia				2		✓	x	x	x									PRJNA396809	[S8]
PYCC 6721	CBS 420; VKM Y-482	DAIRY	Cheese	Italy				1		✓	x	x	x	x	0.29	1.10	0.32	1.10	0.37	1.09	0.05	PRJEB36095	[S6]
PYCC 6720	CBS 437, NRRL Y-1545	DAIRY	Stracchino Cheese	Italy				2		✓	x	x	x	x	0.31	1.18	0.35	1.24	0.31	1.01	-0.04	PRJEB36095	[S6]
A6		AFRICAN BEER	Sorghum beer	Northern Ghana, Africa				1		x	✓	✓	x	--	--	--	NO GROWTH		0.45	0.87	--	PRJEB63270	This sudy
A12		AFRICAN BEER	Sorghum beer	Northern Ghana, Africa				1		x	✓	✓	x	--	--	--	NO GROWTH		0.45	1.11	--	PRJEB63270	This sudy
CLIB 653		AFRICAN BEER	Beer leaven for Bili Bili beer, brewery	Chad				1		x		✓	x									ERP014555	[S1]
NIGF1		AFRICAN BEER	Burukutu	Akure, Nigeria				1		x		✓	x									PRJNA680387	[S9]
N26-1-10:7-5(a)		AFRICAN BEER	Maize dough (kenkey)	Southern Ghana				1		x	✓	✓	✓									ERP014555	[S1]
N34:2-4(a)		AFRICAN BEER	Maize dough (kenkey)	Southern Ghana				1		x	✓	✓	✓									ERP014555	[S1]
CBS 4454		AFRICAN BEER	Kaffir beer	South Africa				1		x	✓	✓	x									ERP014555	[S1]
CBS 4456		AFRICAN BEER	Kaffir beer	South Africa				1		x	✓	✓	x	✓	--	1.25	0.24	1.33	0.35	1.07	0.12	PRJEB36095	[S6]
SAF2		AFRICAN BEER	Sorghum beer	Shakadza, Limpopo province, South Africa				1		x	✓	✓	x									PRJNA680387	[S9]
SAN16		AFRICAN BEER	Guava tree	Tshikweta, Limpopo province, South Africa				1		x	✓		x									PRJNA680387	[S9]
DBVPG 10158		MO	Quecus cerris (soil)	Riserva Naturale Lucciolabella/Chianciano (SI) 2012				1		x	✓	✓	x									PRJEB36095	[S6]
EXF 7197		MO	Quercus sp.	Montenegro				1		x	✓	✓	x	✓	--	1.43	0.24	1.29	0.56	1.18	0.32	ERP014555	[S1]
EXF 7200		MO	Quercus robur	Montenegro, on the road from Durmitor to Niksic (Jasenovo polje)				1			✓	✓	x	✓	--	1.38	0.27	1.49	0.48	1.09	0.22	PRJEB7601	[S5]
HUN9		MO	Oak	Hungarian isolate provided by Eladio Barrio				1			✓	✓	x									PRJEB7601	[S5]
N19-1C		MO	Exudate of Quercus sp.	Gelendzhik region, Caucasus, Russia				1		x	✓	✓	x									ERP014555	[S1]
ZP 1216		MO	Soil from Fig tree	Quinta do Grilo, Mêda, Guarda, Portugal				1		x	✓	✓	x	✓	--	1.15	0.06	0.85	0.45	1.15	0.39	PRJEB59588	[S10]
ZP 568		MO	Q. pyrenaica	Castelo de Vide, Portugal				1		x	✓	✓	x									PRJEB7601	[S5]
ZP 850		MO	Q. ilex	Alconorales Natural Park, Andaluzia, Spain				1			✓	✓	x									PRJEB7601	[S5]
ZP 541		MO	Fagus sylvatica	Adagoi, Portugal				1			✓	✓	x	✓	--	1.37	0.27	1.48	0.45	1.20	0.18	PRJEB7601	[S5]
ZP 736		MO	Rotten figs	Caratão, Abrantes, Portugal				1			✓	✓	x									PRJEB7601	[S5]
UFMG-CM-Y263	C41	WB3	Tapirira guianensis	Tocantins, Brazil							✓	x	✓	x	0.42	1.30	0.40	1.23	0.50	1.11	0.10	PRJEB11698	[S11]
UFMG-CM-Y642	TOC 1365	WB3	Tapirira guianensis	Tocantins, Brazil				2		✓	✓	x	✓	x	0.43	1.27	0.40	1.23	0.49	1.09	0.09	PRJEB11698	[S11]
UFMG-CM-Y641	TOC 1345	WB3	Tapirira guianensis	Tocantins, Brazil							✓	x	✓	x	0.43	1.25	0.43	1.23	0.49	1.13	0.06	PRJEB11698	[S11]
NRRL YB-908		WB3	Wild cherry tree gum	Wickerham, NRRL GM				1		x	✓	✓	x	✓	--	1.36	0.27	1.36	0.51	1.03	0.25	PRJEB36095	[S6]
LCBG-3D2	CPM	MA	Agave spp. fermentation during rustic mezcal production	Tamaulipas, Mexico				1		x	✓	✓	x									ERP014555	[S1]
LCBG-3D6	CPI	MA	Agave spp. fermentation during rustic mezcal production	Tamaulipas, Mexico				1		x	✓	✓	x									ERP014555	[S1]
LCBG-3Y8	CPR	MA	Agave spp. fermentation during rustic mezcal production	Tamaulipas, Mexico				1		x	✓	✓	x									ERP014555	[S1]
CEY 619	BDT	FG	Human feces	French Guiana				1		x	✓	✓	x	✓	--	1.27	0.24	1.42	0.44	1.27	0.20	ERP014555	[S1]
HE004	BCC	FG	Human feces	French Guiana				1		x	✓	✓	x									ERP014555	[S1]
HE012	BCL	FG	Human feces	French Guiana				1			✓	✓	x	✓	--	1.15	0.20	1.47	0.45	1.26	0.25	ERP014555	[S1]
PYCC 4455	CBS 1171; NRRL Y-12632	BEER 1	brewer's top yeast	Rotterdam				1		x	✓	✓	x									PRJEB36095	[S6]
TUM 174		BEER 1	Alt Beer top fermenting yeast S. cerevisiae					1		x	✓	✓	x									ERS1108621	[S12]
TUM 308		BEER 1	Alt Beer top fermenting yeast S. cerevisiae					1		x	✓	✓	x									ERS1108619	[S12]
TUM 513		BEER 1	California Ale					1		x	✓	✓	x									ERS1108618	[S12]

TableS1

TUM 175		BEER 1	Wheat Beer top fermenting yeast S. cerevisiae					1		x	✓	✓	x									ERS1108615	[S12]
TUM 381		BEER 1	Trappist / Abbey Style					1		x	✓	✓	x									ERS1108612	[S12]
TUM 507		BEER 1	Ale / Wheat Beer					1		x	✓	✓	x									ERS1108616	[S12]
TUM 210		BEER 1	British Ale / Stout					1		x	✓	✓	x									ERS1108631	[S12]
TUM 211		BEER 1	Ale & Stout top fermenting yeast S. cerevisiae					1		x	✓	✓	x									ERS1108632	[S12]
TUM 503		BEER 1	California Ale					1		x	✓	✓	x									ERS1108626	[S12]
AP 13.1		BREAD	Fermented sourdough	Cinfães, Portugal				1		x	✓	✓	x	✓	--	1.31	0.26	1.47	0.50	1.08	0.23	PRJEB24932	[S7]
PYCC 5320		BREAD	homemade corn and rye bread dough	Braga, Portugal									x	✓	--	1.30	0.26	1.46	0.45	1.08	0.19	PRJEB36095	[S6]
MorrocoBreadG17	MBG17	BREAD	Baker's yeast	Morrocco				1		x	✓	✓	x									SRR403240	[S13]
MTF3642		BREAD	Sourdough	Namur, Belgium				1			✓		x									PRJEB36058	[S14]
MTF3992		BREAD	Sourdough	Moddizzosu, Italy				1					x									PRJEB36058	[S14]
PYCC 4226		BREAD	Baker's yeast	commercial baker's yeast (Fermipan)				1		x	✓	✓	x	✓	--	1.41	0.24	1.46	0.40	1.15	0.16	ERS1108635	[S12]
Platinum		BREAD	Baker's yeast	USA				1		x	✓	✓	x									ERS1108633	[S12]
BJ2		CHN VIII	Apple, orchard	Shunyi, Beijing				1		x	✓	✓	x									PRJNA396809	[S8]
BJ10		CHN VIII	Bark of Ulmus sp., secondary forest	Wuling Mountain, Beijing				1		x	✓	✓	x									PRJNA396809	[S8]
SR23		CHN VIII	Fagus hayatae	Yilan, Datong, Taiwan				1		x	✓	✓	x									PRJNA755173	[S15]
S16		CHN VIII	Hedychium coronarium	Taipei, Nangang, Taiwan				1		x	✓	✓	x									PRJNA755173	[S15]
MTF2546	CQC	COCOA	Cocoa bean fermentation	West Africa				1			✓		x									ERP014555	[S1]
MTF2551	CQH	COCOA	Cocoa bean fermentation	West Africa				1		x	✓	✓	☒									ERP014555	[S1]
MTF2552	CQI	COCOA	Cocoa bean fermentation	West Africa				1			✓	✓	x									ERP014555	[S1]
MTF2548	CQE	COCOA	Cocoa bean fermentation	West Africa				1		x		✓	x									ERP014555	[S1]
CBS 1598		SAKE	sake moto	Japan				1		x	✓	✓	x									ERP014555	[S1]
PYCC 8194	CBS 435, AQN	SAKE	sake moto	Tokyo				1		x	✓	✓	x	✓	--	1.35	0.24	1.42	0.35	1.237	0.11	ERS1108637	[S12]
Kyokai 7	CBS 6412, AFM	SAKE	Japanese sake breweries	Japan				1		x	✓	✓	x									PRJNA45827	S16
RG02		SAKE	Industrial	Taiwan				1		x	✓	✓	x									PRJNA755173	[S15]
HQ1.1		SAKE	Huangjiu (rice wine) starter with Fermented monascus	Gutian, Fujian				1			✓	✓	✓									PRJNA396809	[S8]
RG10		SAKE	Fermentation	Pingtung, Taiwan				1		x	✓	✓	x									PRJNA755173	[S15]
SR24		SAKE	Fermentation	Taipei, Taiwan				1		x	✓	✓	x									PRJNA755173	[S15]
SAN8		Mauritius/South Africa	Lychee tree	Tshikweta Limpopo province, South Africa				1		x	✓	✓	x									PRJNA680387	[S9]
SAN9		Mauritius/South Africa	Lychee tree	Tshikweta Limpopo province, South Africa				1		x	✓	✓	x									PRJNA680387	[S9]
MAUN3		Mauritius/South Africa	Dracaema reflexa (tree)	Casela, Mauritius						x	✓	✓	x									PRJNA680387	[S9]
MAUN5		Mauritius/South Africa	Dracaema reflexa (tree)	Casela, Mauritius				1		x	✓	✓	x									PRJNA680387	[S9]
MAUF1		Mauritius/South Africa	Mango Juice	Quatre Bornes, Mauritius				1		x	✓	✓	x									PRJNA680387	[S9]
MAUF2		Mauritius/South Africa	Mango Juice	Quatre Bornes, Mauritius				1		x	✓	✓	x									PRJNA680387	[S9]
NRRL Y-5508	PYCC 8029	PHILIPPINES	coconut pod	Arguelles, Manila, Philippines				1		x	✓	✓	x	--	--	--	NO GROWTH		0.41	1.12	--	PRJEB36095	[S6]
NRRL Y-6297	PYCC 8030	PHILIPPINES	Coconut tuba, Phillipines	Hipolito, NIST, Manila, Phillipines				1		x	✓	✓	x	--	--	--	NO GROWTH		0.38	1.12	--	PRJEB36095	[S6]
YJM 1479	SACE_YDE; NRRL Y-6297	PHILIPPINES	Coconut water	D. Hipolito, NIST, Manila, Philippines				1			✓	✓	x									PRJNA189929	[S17]
TBRC 9268		PHILIPPINES	Nypa palm fruit stem sap	Pak Phanang, Nakhon Si Thammarat, Thailand				1		x	✓	✓	x	✓	--	1.30	0.29	1.26	0.56	0.90	0.27	PRJEB63270	This sudy
TBRC 9269		PHILIPPINES	Nypa palm fruit stem sap	Kantang, Trang, Thailand				1		x	✓	✓	x	✓	--	1.36	0.25	1.37	0.56	1.04	0.31	PRJEB63270	This sudy
TBRC 9270		PHILIPPINES	Toddy palm inflorescence sap	Ban Lat, Phetchaburi, Thailand				1		x	✓	✓	x	✓	--	1.20	0.27	1.31	0.61	0.86	0.34	PRJEB63270	This sudy
TBRC 9271		PHILIPPINES	Toddy palm inflorescence sap	Sankhaburi, Chai Nat, Thailand				1		x			x	✓	--	1.26	0.28	1.31	0.63	0.95	0.35	PRJEB63270	This sudy
TBRC 9266		PHILIPPINES	Coconut inflorescence sap	Bang Khont, Sa Mut Songkhram, Thailand				1		x	✓	✓	x	✓	--	1.12	0.27	1.22	0.66	0.70	0.40	PRJEB63270	This sudy
TBRC 9267		PHILIPPINES	Coconut inflorescence sap	Mueang, Samut Songkhram, Thailand				1		x	✓	✓	x	✓	--	1.33	0.17	1.41	0.52	1.03	0.34	PRJEB63270	This sudy
PD41A		TW_Mosaic	Pinus sp.	Island, Dongsha,Taiwan				1		x	✓	✓	✓									PRJNA755173	[S15]
S8		TW_Mosaic	Quercus stenophylloides	Yilan, Yuanshan, Taiwan				1		x	✓	✓	x									PRJNA755173	[S15]
XI07		TW_Mosaic	Stachyurus himalaicus	Yilan, Datong, Taiwan				1			✓		x									PRJNA755173	[S15]
PD04A		TW5	Quercus glauca	Taipei, Daan, Taiwan				1		x	✓	✓	x									PRJNA755173	[S15]
PD09A		TW5	Quercus glauca	Taipei, Daan, Taiwan				1		x	✓	✓	x									PRJNA755173	[S15]
PD20A		TW5	Quercus glauca	Taipei, Daan, Taiwan				1		x	✓	✓	x									PRJNA755173	[S15]
PD14A		TW5	Quercus glauca	Taipei, Daan, Taiwan				1		x	✓	✓	x									PRJNA755173	[S15]
ZP 651		NA & JP	Q. acutissima	Chiba Perf., Japan				1		x	✓	✓	x	✓	--	1.40	0.24	1.35	0.49	1.08	0.25	PRJEB7601	[S5]
ZP 656		NA & JP	Q. acuta	Chiba Perf., Japan				1		x	✓	✓	x	✓	--	1.36	0.29	1.31	0.53	1.07	0.24	PRJEB7601	[S5]
ZP 652		NA & JP	Q. acutissima	Chiba Perf., Japan				1		x	✓		x	✓	--	1.41	0.27	1.26	0.47	1.11	0.20	PRJEB7601	[S5]
ZP 779		NA & JP	Q. acutissima	Hirusen highland, Okayama Perf., Japan				1		x	✓	✓	x	✓	--	1.38	0.28	1.45	0.46	1.12	0.18	PRJEB7601	[S5]
ZP 781		NA & JP	Q. serrata	Hirusen highland, Okayama Perf., Japan				1		x	✓	✓	x	✓	--	1.37	0.28	1.50	0.45	1.25	0.17	PRJEB7601	[S5]
PD30A		CHN VI/VII.2	Castanopsis carlesii	Taitung, Beinan,Taiwan				1			✓	✓	x									PRJNA755173	[S15]
PD32A		CHN VI/VII.2	Castanopsis carlesii	Taitung, Beinan,Taiwan				1		x	✓	✓	x									PRJNA755173	[S15]
BJ28		CHN VI / CHN VII						1		x	✓	✓	x									PRJNA396809	[S8]
FJ12		CHN VI / CHN VII						1		x	✓	✓	x									PRJNA396809	[S8]
HLJ2		CHN VI / CHN VII						1		x	✓	✓	x									PRJNA396809	[S8]
TBRC 618			Moss	Tong Pha Phum, Kanchanaburi, Thailand				1		x	✓	✓	x	✓	--	1.33	0.27	1.41	0.49	1.01	0.22	PRJEB63270	This sudy
XJ1								1		x	✓	✓	x									PRJNA396809	[S8]
LY0200		TW4	Physciaceae sp.	Taichung, Heping, Taiwan				1		x	✓	✓	x									PRJNA755173	[S15]
LY0197		TW4	Parmeliaceae sp.	Taichung, Heping, Taiwan				1		x	✓	✓	x									PRJNA755173	[S15]
LY0177		TW4	Peltigeraceae sp.	Taichung, Heping, Taiwan				1		x	✓	✓	x									PRJNA755173	[S15]
H416-8		TW4	Actinodaphne sp.	Yilan, Datong, Taiwan				1		x	✓	✓	x									PRJNA755173	[S15]
N3.00-7A	CFG	FAR EAST RUSSIA	Grape berries of wild Vitus amurensis	Botanical garden, Blagoveshchensk, Russia				1		x	✓	✓	x									ERP014555	[S1]
N163.01-5A	CDI	FAR EAST RUSSIA	Exudate of Quercus mongolica	Botanical garden, Blagoveshchensk, Russia				1		x	✓	✓	x									ERP014555	[S1]
N22.00-5D	CDF	FAR EAST RUSSIA	Fruit of Grataegus dachurica	Botanical garden, Blagoveshchensk, Russia				1		x	✓	✓	x									ERP014555	[S1]
N159.01-2B	CDH	FAR EAST RUSSIA	Exudate of Quercus mongolica	Botanical garden, Blagoveshchensk, Russia				1		x	✓	✓	x									ERP014555	[S1]
TBRC 3578			Lichen	Chom Tong, Chiangmai, Thailand				1		x	✓	✓	✓	✓	--	1.42	0.31	1.45	0.53	1.13	0.22	PRJEB63270	This sudy
TBRC 3589			Insect frass	Chom Tong, Chiangmai, Thailand				1		x	✓	✓	✓	✓	--	1.42	0.33	1.46	0.53	1.20	0.20	PRJEB63270	This sudy
SD1								1		x	✓	✓	x									PRJNA396809	[S8]
SX10								1		x	✓	✓	x									PRJNA396809	[S8]
S11		CHN VI / CHN VII	Quercus glauca	Yilan, Yuanshan, Taiwan				1		x	✓	✓	x									PRJNA755173	[S15]
S12		CHN VI / CHN VII	Castanopsis indica	Yilan, Yuanshan, Taiwan				1		x	✓	✓	x									PRJNA755173	[S15]
XI02		CHN VI / CHN VII	Koelreuteria elegans subps. Formosana	New Taipei, Shiding, Taiwan				1		x	✓	✓	x									PRJNA755173	[S15]
S7		CHN VI / CHN VII	Mallotus paniculatus	Taipei, Nangang, Taiwan				1		x	✓	✓	x									PRJNA755173	[S15]
PD27A			Broussonetia papyrifera	Hualien, Fuli, Taiwan				1		x	✓	✓	✓									PRJNA755173	[S15]
PD40A		TW3	Quercus pachyloma	Nantou, Puli,Taiwan				1		x	✓	✓	x									PRJNA755173	[S15]

TableS1

SR28		TW3	Castanopsis fargesii	Nantou, Yuchih, Taiwan	1	x	✓	✓	✓									PRJNA755173	[S15]
RG06		TW3	Ardisia cornudentata subsp. morrisonensis	Pingtung, Majia, Taiwan	1	x	✓	x	✓									PRJNA755173	[S15]
SR26		TW3	Quercus glauca	Nantou, Puli, Taiwan	1	x	✓	x	✓									PRJNA755173	[S15]
TBRC 3587		THAI I	Insect frass	Nopphitam, Nakhon Si Thammarat, Thailand		✓	✓	x	✓	x	0.37	1.35	0.39	1.34	0.50	1.11	0.11	PRJEB63270	This sudy
TBRC 617		THAI I	Moss	Tong Pha Phum, Kanchanaburi, Thailand	1	✓	✓	x	✓	x	0.40	1.39	0.42	1.25	0.50	1.15	0.08	PRJEB63270	This sudy
TBRC 3978		THAI I	Mushroom	Khlong Lan, Kamphaeng Phet, Thailand	1	x	✓	✓	✓	✓	--	1.43	0.28	1.48	0.54	1.15	0.26	PRJEB63270	This sudy
TBRC 3977		THAI I	Mushroom	Khlong Lan, Kamphaeng Phet, Thailand		✓	✓	x	x	x	0.44	1.38	0.43	1.31	0.53	1.11	0.09	PRJEB63270	This sudy
TBRC 616		THAI I	Insect frass	Tong Pha Phum, Kanchanaburi, Thailand		✓	✓	x	✓	x	0.39	1.36	0.40	1.34	0.49	1.17	0.09	PRJEB63270	This sudy
TBRC 3588			Mushroom	Khlong Thom, Krabi, Thailand	1	x	✓	✓	✓	✓	--	1.31	0.32	1.43	0.57	1.09	0.25	PRJEB63270	This sudy
HN16	BAP		Soil	Wuzhi Mountain, Hainan province, China	1	x	✓	✓	✓									ERP014555	[S1]
HN15	BAN		Rotten wood	Wuzhi Mountain, Hainan province, China	1	x	✓	✓	✓									ERP014555	[S1]
S4		TW2	Lemmaphyllum carnosum var. microphyllum	Taipei, Nangang, Taiwan	1	x	✓	✓	✓									PRJNA755173	[S15]
SR11		TW2	Quercus tarokoensis	Yilan, Yuanshan, Taiwan	1	x	✓	✓	✓									PRJNA755173	[S15]
SR16		TW2	Lithocarpus hancei	Yilan, Yuanshan, Taiwan	1	x	✓	✓	✓									PRJNA755173	[S15]
XI06		TW2	Ulmus uyematsui	Yilan, Datong, Taiwan	1	x	✓	✓	✓									PRJNA755173	[S15]
UFMG-CM-Y455	C45	WB1 - CHN V	Tapirira guianensis	Tocantins, Brazil	1	x	✓	✓	✓	✓	--	1.42	0.29	1.48	0.54	1.15	0.26	PRJEB11698	[S11]
UFMG-CM-Y650	TOC1364	WB1 - CHN V	Tapirira guianensis	Tocantins, Brazil	1	x	✓	✓	✓	✓	--	1.30	0.24	1.45	0.43	1.18	0.19	PRJEB11698	[S11]
UFMG-CM-Y461	C145	WB1 - CHN V	Mushroom	Roraima, Brazil	1	x	✓	✓	✓	✓	--	1.41	0.29	1.49	0.53	1.16	0.24	PRJEB11698	[S11]
CLQCA 20-156	CCC	WB1 - CHN V	Flower (Gesneriaceae sp.)	Yasuni, Orellana	1	x	✓	✓	✓									ERP014555	[S1]
CLQCA 20-314	CCH	WB1 - CHN V	Termite mound	Yasuni, Orellana	1	x	✓	✓	✓									ERP014555	[S1]
UFMG-CM-Y5863		WB1 - CHN V	Wood in decomposition (Hybrid S.cer x S.par)	Parque Nacional do Iguaçu - Foz do Iguaçu- PR	1	x ✓	✓	✓	✓	x	0.33	1.33	0.34	1.38	0.48	1.16	0.13	PRJEB63270	This sudy
UFMG-CM-Y2163		WB1 - CHN V	Wood in decomposition	Parque Estadual do Rio Doce - MG	2	✓	✓	x	✓	x	0.40	1.24	0.38	1.27	0.49	1.16	0.11	PRJEB63270	This sudy
UFMG-CM-Y-646	TOC 1322.1	WB1 - CHN V	Tapirira guianensis	Tocantins, Brazil	2	✓	✓	x	✓	x	0.42	1.32	0.4	1.3	0.55	1.13	0.14	PRJEB11698	[S11]
UFMG-CM-Y-647	TOC 1325.1	WB1 - CHN V	Tapirira guianensis	Tocantins, Brazil	2		✓	x	✓									PRJEB11698	[S11]
TBRC 3580			Lichen	Phu Ruea, Loei, Thailand	1	x	✓	✓	x	✓	--	1.43	0.25	1.45	0.55	1.05	0.30	PRJEB63270	This sudy
CBS 403	PYCC 4571	WEST AFRICA	Ginger beer from Zingiber officinale Type of *S. lindneri*	West Africa	1	x	✓	✓	☒				NO GROWTH		0.33	1.47	--	ERP014555	[S1]
YJM 1248	DBVPG 6044, NRRLY 1546	WEST AFRICA	Bili wine	West Africa	1	x	✓	✓	☒									PRJNA189888	[S17]
NRRL Y-12633	PYCC 2542; CBS 400	WEST AFRICA	Palm wine (Y12 strain in Fay and Benevides 2005)	Ivory Coast	1	x	✓	✓	☒									PRJEB36095	[S6]
CBS 1419		WEST AFRICA	Palm wine from Elaeis guineensis	Ghana (H.A. Dade, Feb 1932)	1	x	✓	✓	x									PRJEB63270	This sudy
CBS 7903	NCYC 761	WEST AFRICA	Palm wine	Nigeria	1	x	✓	✓	x									PRJEB36095	[S6]
HN10	BAM	CHN III	Rotten wood	Wuzhi Mountain, Hainan province, China	2	✓	x	x	✓	x	0.37	1.24	0.40	1.28	0.43	1.08	0.03	ERP014555	[S1]
HN19		CHN III	Bark from tree in Fagaceae family	Wuzhi Mountain, Hainan province, China	2	✓	x	x	✓	x	0.35	1.21	0.38	1.29	0.40	1.15	0.02	ERP014555	[S1]
TBRC 614		THAI II	Insect frass	Tong Pha Phum, Kanchanaburi, Thailand	1	x	✓	✓	✓	✓	--	1.36	0.29	1.43	0.53	1.10	0.24	PRJEB63270	This sudy
TBRC 615		THAI II	Moss	Tong Pha Phum, Kanchanaburi, Thailand	1	x	✓	✓	✓	✓	--	1.39	0.29	1.40	0.52	1.07	0.23	PRJEB63270	This sudy
TBRC 3581		THAI II	Tree exudate	Sangkhlaburi, Kanchanaburi, Thailand	1	x	✓	✓	✓	✓	--	1.33	0.31	1.32	0.51	1.08	0.20	PRJEB63271	This sudy
GT39.1		CHN X	Bark, primeval forest	Gutian Mountain, Zhejiang	1	x	✓	✓	✓									PRJNA396809	[S8]
JXXY1.1		CHN X	Bark, primeval forest	Xiangxiyuan, Shennongjia, Hubei	1	x	✓	✓	✓									PRJNA396809	[S8]
ZP 1273		CHN X		New Caledonia	1	x	✓	✓	x									PRJEB36095	[S6]
UWOPS 03-433.3	BMB	MALAYSIA	Nectar of Bertram palm	Malaysia	1	x	✓	✓	✓	✓	--	1.34	0.21	1.41	0.46	1.09	0.24	ERS1108647	[S12]
UWOPS 03-461.4	AKM	MALAYSIA	Nectar of Bertram palm	Malaysia	1	x	✓	✓	✓	✓	--	1.31	0.22	1.37	0.45	1.10	0.24	SGRP2	[S18]
YJM 1447	SACE_YCY	MALAYSIA	Bertram palm	Malaysia	1	x	✓	✓	✓									PRJNA189923	[S17]
TBRC 3614			Lichen	Tha Wang Pha, Nan, Thailand	1	x	✓	✓	✓	✓	--	1.35	0.32	1.43	0.53	1.26	0.21	PRJEB63270	This sudy
BJ6	BAI	CHN IV	Persimmon	Changping, Beijing, China	1	x	✓	✓	x									ERP014555	[S1]
BJ21		CHN IV	Bark of Carya cathayensis, secondary forest	Dongling Mountain, Beijing	1	x	✓	✓	x									PRJNA396809	[S1]
BJ14		CHN IV	Forest soil, secondary forest	Wuling Mountain, Beijing	1	x	✓	✓	x									PRJNA396809	[S8]
HLJ1		CHN IV	Bark of Quercus mongoLica, secondary forest	Jingbo lake, Heilongjiang	1	x	✓	✓	x									PRJNA396809	[S8]
N51-1A	CDL	CHN IV	Exudate of Quercus mongolica	Vladivostok, Russian Far East	1	x	✓	✓	x									ERP014555	[S1]
HN6	BAL	CHN I	Rotten wood	Wuzhi Mountain, Hainan province, China	1	x	✓	x	x									ERP014555	[S1]
HN2		CHN I	Rotten wood, primeval forest	Diaoluo Mountain, Hainan	1	x	✓	x	✓									PRJNA396809	[S8]
SX1	BAG	CHN II	Bark from tree in Fagaceae family	Qinling Mountain, Shaanxi province, China	1	x	✓	✓	x									ERP014555	[S1]
SX3	BAH	CHN II	Bark from Carya sp.	Qinling Mountain, Shaanxi province, China	1	x	✓	✓	x									ERP014555	[S1]
SX8		CHN II	Bark of Quercus fabri, primeval forest	Qinling Mountain, Shaanxi	1	x	✓	✓	x									PRJNA396809	[S8]
JXXY16.1		CHN IX	Bark, primeval forest	Xiangxiyuan, Shennongjia, Hubei	1	x	✓	✓	x									PRJNA396809	[S8]
XXYS1.4		CHN IX	Bark, primeval forest	Xiangxiyuan, Shennongjia, Hubei	1		✓	✓	x									PRJNA396809	[S8]
XXY30L.2		CHN IX	Bark, primeval forest	Xiangxiyuan, Shennongjia, Hubei	1		✓	✓	x									PRJNA396809	[S8]
EN14S01		TAIWAN 1	Soil	Sinyi, Nantou, Taiwan	1	x	✓	✓	x									ERP014555	[S1]
XI04		TAIWAN 1	Itea parviflora	Yilan, Datong, Taiwan	1		✓	✓	x									PRJNA755173	[S15]
SPA0344		TAIWAN 1	Lithocarpus kawakamii	Yilan, Datong, Taiwan	1		✓	✓	x									PRJNA755173	[S15]

GAL Cluster and *PGM1* versions

Reference version - *S. cerevisiae*: Reference

Alternative version - *S. cerevisiae*: CER - A1; CER - A2; CER - A3

Alternative version - *S. paradoxus*: PAR - A1

Recombiant version - *S. cerevisiae* reference x *S. cerevisiae* alternative

Gal4-binding site promotor region of *PGM1*

✓ Gal4-binding site present

x Gal4-binding site absent

Mig1-biding site in the promoter reigion of *GAL* genes

x No binding or low affinity Mig1-binding site in the promoter region of *GAL4* or *GAL1*/*GAL10*

✓ Mig1-binding site in the promoter region of GAL4 or *GAL1*/*GAL10*

Phenotype

✓ Diauxic Growth in YNB 1X + 1% Glucose + 1% Galactose

x No Diauxic Growth in YNB 1X + 1% Glucose + 1% Galactose

MEL

x *MEL* gene Absent

✓ *MEL* gene Present and functional

☒ *MEL* gene Present but not functional

No data available

Supplementary Table 2. *S. paradoxus* genomes surveyed for the presence of *GAL* alternative versions, related to Figure 1.

Species	Population	Strain	*GAL7*	*GAL10*	*GAL1*	*GAL2*	Accession number
S. paradoxus	EUROPE	CBS 432 [NT]					PRJEB7245
S. paradoxus	EUROPE	Q59					SGRP
S. paradoxus	EUROPE	Z1					SGRP
S. paradoxus	FAR EAST	N-44					PRJEB7245
S. paradoxus	FAR EAST	JL-WQ14-1					PRJNA761003
S. paradoxus	FAR EAST	XZ-98-1-1					PRJNA761003
S. paradoxus	FAR EAST	HB-XS21-2					PRJNA761003
S. paradoxus	FAR EAST	HB-XS1-1					PRJNA761003
S. paradoxus	FAR EAST	HB-SNJ2a					PRJNA761003
S. paradoxus	FAR EAST	HB-MY15-2					PRJNA761003
S. paradoxus	FAR EAST	HB-XS3-1					PRJNA761003
S. paradoxus	FAR EAST	HB-XXY4-1					PRJNA761003
S. paradoxus	FAR EAST	HB-XS18-1					PRJNA761003
S. paradoxus	FAR EAST	JL-CB13-1					PRJNA761003
S. paradoxus	FAR EAST	BJ-DLS32-27					PRJNA761003
S. paradoxus	FAR EAST	SN-TTH1-1					PRJNA761003
S. paradoxus	FAR EAST	HB-MY10-3					PRJNA761003
S. paradoxus	FAR EAST	SN-TTS3-10					PRJNA761003
S. paradoxus	FAR EAST	SN-HZZ24-1					PRJNA761003
S. paradoxus	FAR EAST	SN-ZZ18-9					PRJNA761003
S. paradoxus	CHINA	BJ-DLS32-26					PRJNA761003
S. paradoxus	CHINA	BJ-DLS60-3					PRJNA761003
S. paradoxus	CHINA	BJ-DLS2-1					PRJNA761003
S. paradoxus	CHINA	LL2012_001					PRJNA277692
S. paradoxus	NORTH AMERICA	YPS 138					PRJEB7245
S. paradoxus	NORTH AMERICA	95-7-1D					PRJNA277692
S. paradoxus	NORTH AMERICA	LL2013_055					PRJNA277692
S. paradoxus	NORTH AMERICA	LL2013_030					PRJNA277692
S. paradoxus	NORTH AMERICA	LL2013_043					PRJNA277692
S. paradoxus	NORTH AMERICA	LL2013_051					PRJNA277692
S. paradoxus	NORTH AMERICA	LL2012_030					PRJNA277692
S. paradoxus	NORTH AMERICA	LL2012_022					PRJNA277692
S. paradoxus	NORTH AMERICA	LL2011_004					PRJNA277692
S. paradoxus	NORTH AMERICA	LL2011_012					PRJNA277692
S. paradoxus	NORTH AMERICA	LL2013_200					PRJNA277692
S. paradoxus	NORTH AMERICA	LL2013_205					PRJNA277692
S. paradoxus	NORTH AMERICA	LL2012_011					PRJNA277692
S. paradoxus	NORTH AMERICA	LL2012_016					PRJNA277692
S. paradoxus	NORTH AMERICA	LL2012_018					PRJNA277692
S. paradoxus	NORTH AMERICA	LL2012_020					PRJNA277692
S. paradoxus	NORTH AMERICA	LL2012_027					PRJNA277692
S. paradoxus	NORTH AMERICA	LL2013_010					PRJNA277692
S. paradoxus	NORTH AMERICA	LL2013_012					PRJNA277692
S. paradoxus	NORTH AMERICA	LL2013_183					PRJNA277692
S. paradoxus	NORTH AMERICA	LL2012_014					PRJNA277692

Reference version

Supplemental References List

[S1] Peter, J., De Chiara, M., Friedrich, A., Yue, J.-X., Pflieger, D., Bergström, A., Sigwalt, A., Barre, B., Freel, K., Llored, A. (2018). Genome evolution across 1,011 *Saccharomyces cerevisiae* isolates. Nature *556*, 339–344. 10.1038/s41586-018-0030-5.

[S2] Almeida P, Barbosa R, Bensasson D, Gonçalves P, Sampaio JP. (2017). Adaptive divergence in wine yeasts and their wild relatives suggests a prominent role for introgressions and rapid evolution at noncoding sites. Mol. Ecol. *26*, 2167-2182. doi: 10.1111/mec.14071.

[S3] Novo M, Bigey F, Beyne E, Galeote V, Gavory F, Mallet S, Cambon B, Legras JL, Wincker P, Casaregola S, Dequin S. (2009). Eukaryote-to-eukaryote gene transfer events revealed by the genome sequence of the wine yeast *Saccharomyces cerevisiae* EC1118. PNAS *106*, 16333-16338. https://doi.org/10.1073/pnas.0904673106.

[S4] Borneman AR, Desany BA, Riches D, Affourtit JP, Forgan AH, Pretorius IS, Egholm M, Chambers PJ (2011). Whole-genome comparison reveals novel genetic elements that characterize the genome of industrial strains of *Saccharomyces cerevisiae*. PLoS Genet. *7*, E1001287. https://doi.org/10.1371/journal.pgen.1001287.

[S5] Almeida P, Barbosa R, Zalar P, Imanishi Y, Shimizu K, Turchetti B, Legras JL, Serra M, Dequin S, Couloux A, Guy J, Bensasson D, Gonçalves P, Sampaio JP. (2015). A population genomics insight into the Mediterranean origins of wine yeast domestication. Mol Ecol. *24,* 5412-27. doi: 10.1111/mec.13341.

[S6] Pontes, A., Hutzler, M., Brito, P.H., and Sampaio, J.P. (2020). Revisiting the taxonomic synonyms and populations of *Saccharomyces cerevisiae* – phylogeny, phenotypes, ecology and domestication. Microorganisms *8*, 903.

[S7] Barbosa R, Pontes A, Santos RO, Montandon GG, de Ponzzes-Gomes CM, Morais PB, Gonçalves P, Rosa CA, Sampaio JP. (2018). Multiple rounds of artificial selection promote microbe secondary domestication – the case of cachaça yeasts. GBE *10*, 1939-1955. https://doi.org/10.1093/gbe/evy132.

[S8] Duan SF, Han PJ, Wang QM, Liu WQ, Shi JY, Li K, Zhang XL, Bai FY. (2018). The origin and adaptive evolution of domesticated populations of yeast from Far East Asia. Nature communications. *9*.1: 2690. doi: 10.1038/s41467-018-05106-7.

[S9] Han, D.Y., Han, P.J., Rumbold, K., Koricha, A.D., Duan, S.F., Song, L., Shi, J.Y., Li, K., Wang, Q.M., and Bai, F.Y. (2021). Adaptive Gene Content and Allele Distribution Variations in the Wild and Domesticated Populations of *Saccharomyces cerevisiae*. Front Microbiol *12*. 10.3389/fmicb.2021.631250.

[S10] Paraíso F., Pontes A., Neves J. , Lebani K., Hutzler M. , Zhou,N. , Sampaio, JP. (2023). Do microbes evade domestication? - Evaluating potential ferality among diastatic *Saccharomyces cerevisiae*, Food Microbiol. , *115*, 0740-0020, https://doi.org/10.1016/j.fm.2023.104320.

[S11] Barbosa, R., Almeida, P., Safar, S.V.B., Santos, R.O., Morais, P.B., Nielly-Thibault, L., Leducq, J.B., Landry, C.R., Gonçalves, P., Rosa, C.A., et al. (2016). Evidence of natural hybridization in Brazilian wild lineages of *Saccharomyces cerevisiae*. Genome Biol Evol, *8*, 317–329. 10.1093/gbe/evv263.

[S12] Gonçalves M, Pontes A, Almeida P, Barbosa R, Serra M, Libkind D, Hutzler M, Gonçalves P, Sampaio JP. (2016). Distinct domestication trajectories in top-fermenting beer yeasts and wine yeasts. Curr Biol, *26*, 2750-2761. doi: 10.1016/j.cub.2016.08.040.

[S13] Dunn B, Richter C, Kvitek DJ, Pugh T, Sherlock G. (2012). Analysis of the *Saccharomyces cerevisiae* pan-genome reveals a pool of copy-number variants distributed in diverse yeast strains from differing industrial environments. Genome Res. *22*, 908-924. doi: 10.1101/gr.130310.111.

[S14] Bigey F, Segond D, Friedrich A, Guezenec S, Bourgais A, Huyghe L, Agier N, Nidelet T, Sicard D. (2021). Evidence for Two Main Domestication Trajectories in *Saccharomyces cerevisiae* Linked to Distinct Bread-Making Processes, Current Biology, *31*,722-732.e5. https://doi.org/10.1016/j.cub.2020.11.016.

[S15] Lee, T.J., Liu, Y.C., Liu, W.A., Lin, Y.F., Lee, H.H., Ke, H.M., Huang, J.P., Lu, M.Y.J., Hsieh, C.L., Chung, K.F., et al. (2022). Extensive sampling of *Saccharomyces cerevisiae* in Taiwan reveals ecology and evolution of predomesticated lineages. Genome Res *32*, gr.276286.121. 10.1101/GR.276286.121.

[S16] Akao T, Yashiro I, Hosoyama A, Kitagaki H, Horikawa H, Watanabe D, Akada R, Ando Y, Harashima S, Inoue T, Inoue Y, Kajiwara S, Kitamoto K, Kitamoto N, Kobayashi O, Kuhara S, Masubuchi T, Mizoguchi H, Nakao Y, Nakazato A, Namise M, Oba T, Ogata T, Ohta A, Sato M, Shibasaki S, Takatsume Y, Tanimoto S, Tsuboi H, Nishimura A, Yoda K, Ishikawa T, Iwashita K, Fujita N, Shimoi H. (2011). Whole-genome sequencing of sake yeast *Saccharomyces cerevisiae* Kyokai no. 7. DNA Res. *18*, 423-434. doi: 10.1093/dnares/dsr029.

[S17] Strope PK, Skelly DA, Kozmin SG, Mahadevan G, Stone EA, Magwene PM, Dietrich FS, McCusker JH. (2015). The 100-genomes strains, an S. cerevisiae resource that illuminates its natural phenotypic and genotypic variation and emergence as an opportunistic pathogen. Genome Res. *25* (5):762-74. doi: 10.1101/gr.185538.114.

[S18] Bergström, A et al Bergström A, Simpson JT, Salinas F, Barré B, Parts L, Zia A, Nguyen Ba AN, Moses AM, Louis EJ, Mustonen V, Warringer J, Durbin R, Liti G. (2014). A high-definition view of functional genetic variation from natural yeast genomes. Mol Biol Evol *31*, 872–888. doi: 10.1093/molbev/msu037.
